# Factors Contributing to Variability in Longitudinal Pain Scores in Osteoarthritis Randomised Clinical Trials: A Systematic Review and Meta‐Analysis

**DOI:** 10.1002/ejp.70283

**Published:** 2026-05-11

**Authors:** Jakob M. Bentin, Andrea B. Kaaber, Lars Arendt‐Nielsen, Asger R. Bihlet

**Affiliations:** ^1^ NBCD A/S Søborg Denmark; ^2^ Department of Health Science and Technology Center for Neuroplasticity and Pain (CNAP) Aalborg University Aalborg Denmark; ^3^ Department of Gastroenterology & Hepatology Aalborg University Hospital Aalborg Denmark; ^4^ Aalborg University Hospital, Steno Diabetes Center North Denmark Aalborg Denmark

## Abstract

**Background and Objectives:**

Osteoarthritis is a prevalent joint disease causing pain and functional decline, but high variability in patient‐reported treatment outcomes in clinical trials challenges detection of treatment effects. This systematic review aimed to evaluate variability in pain reporting and identify trial characteristics, placebo comparator attributes, and participant clinical characteristics associated with this variability.

**Databases and Data Treatment:**

A systematic review of placebo‐controlled RCTs for pharmacological interventions in knee osteoarthritis was conducted, adhering to PRISMA guidelines and registered at Open Science Framework. MEDLINE and EMBASE databases were searched for trials from 2010 to October 2024. The primary outcome was variability of the Western Ontario and McMaster Universities Osteoarthritis Index (WOMAC) pain subscale. A random‐effects meta‐analysis estimated pooled variability of WOMAC pain change.

**Results:**

From 1318 records, 87 reports involving 36,246 participants were included. The overall mean variability (SD) of WOMAC pain change was 20.7 on a normalised 0–100 scale, with an absolute mean change of 17.3. Variability was linked to trial size, number of sites, study arms, and baseline pain scores. Smaller studies and those with fewer sites showed lower variability. Topical and intra‐articular placebo administration were associated with lower variability. Baseline BMI and pain scores influenced variability, while age and sex did not.

**Conclusions:**

This review highlights significant variability in WOMAC pain reporting in osteoarthritis trials, affecting statistical power and trial design. Key factors influencing variability include trial design, administration route, and participant characteristics. Integrating these variability estimates into sample‐size calculations can enhance the efficiency of future pain trials.

**Significance Statement:**

This first systematic quantification of variability in WOMAC pain across knee osteoarthritis trials shows substantial heterogeneity. Variability directly alters the detectable magnitude of treatment effect and trial sensitivity, and by shifting attention from mean treatment effects to outcome dispersion as a modifiable design element, this critical and insufficiently characterised driver of trial sensitivity provides a new methodological lens for pain trials. The resulting empiric variability benchmarks enables better study design planning, interpretability, and support more efficient development of new therapies.

## 1 Introduction

Osteoarthritis (OA) is a common joint disease in older adults, characterised by joint changes and symptoms such as pain, stiffness, and reduced function (Hunter and Bierma‐Zeinstra 2019). Pain is the principal clinical manifestation and a key driver of therapeutic decision‐making (Deveza and Loeser 2018; Fu et al. 2018; Hunter and Bierma‐Zeinstra 2019). Accordingly, OA research and clinical practice rely heavily on patient‐reported outcome measures, particularly those evaluating pain intensity, physical function, and overall patient global assessment (McAlindon et al. 2015; Neogi 2013; Pham et al. 2004), with the central multidimensional instrument being the Western Ontario and McMaster Universities Osteoarthritis Index (WOMAC) (Bellamy et al. 1988). WOMAC is a validated OA‐specific questionnaire with pain, stiffness, and physical function subscales, where higher scores indicate worse symptoms.

Currently pharmacological treatments primarily provide symptomatic relief. Oral analgesics such as paracetamol/acetaminophen demonstrate modest efficacy (ES 0.18, 95% CI: 0.04–0.33 vs. placebo), whereas nonsteroidal anti‐inflammatory drugs (NSAIDs; e.g., ibuprofen ES 0.44, 95% CI: 0.25–0.63 vs. placebo) (Bannuru, Schmid, et al. 2015) show greater efficacy but are associated with long‐term safety risks (Singh and Triadafilopoulos 1999; Trelle et al. 2011). Intra‐articular corticosteroids provide short‐term relief but may adversely affect joint structure (McAlindon et al. 2017a; Pereira et al. 2025), while hyaluronic acid offers limited clinical benefit (Lo et al. 2012; Pereira et al. 2022). Therefore, there remains a need for therapies that address these limitations.

OA pain is multifactorial, arising from mechanical, inflammatory, and metabolic processes across joint tissues (Deveza and Loeser 2018; Hawker et al. 2008; Miller et al. 2014; Neogi 2013; Sofat et al. 2011). Structural abnormalities such as bone marrow lesions, synovitis, and effusions correlate modestly with symptoms (Yusuf et al. 2011), but structure‐symptom discordance remains (Bedson and Croft 2008). Beyond peripheral nociception, many patients exhibit features of peripheral and central sensitisation, reflecting altered pain processing and heterogenous pain phenotypes (Fu et al. 2018; Schaible et al. 2002).

Evaluating novel treatments in randomised controlled trials (RCTs) is complicated by high variability in patient‐reported outcomes. Statistical power depends on both the between‐group difference and outcome variability, so greater variability reduces power, often requiring larger, costlier trials. Such constraints may discourage the development of new therapies when projected costs exceed available resources. Importantly, OA pain reflects interacting biological, psychological, and social processes that contribute to heterogeneity in clinical presentation and treatment response (Gatchel et al. 2007; Hunter and Bierma‐Zeinstra 2019). Variability may reflect both true variance, driven by temporal fluctuations in pain and contextual influences, and error variance arising from measurement or trial design. Distinguishing between these sources of variability remains challenging and is beyond the scope of the present study.

To date, no systematic reviews have quantified variability in key outcomes such as WOMAC pain, an endpoint commonly accepted by regulatory authorities (EMA CHMP 2010; FDA CDER 2018). This study aims to systematically review comparable OA RCTs to describe within‐trial variability (standard deviation) of patient‐reported pain change scores and explore trial‐level factors associated with these differences.

## 2 Methods

### 2.1 Guidelines

This systematic review follows the PRISMA guidelines for design and reporting.

### 2.2 Registration and Protocol

The protocol was completed and submitted to PROSPERO prior to study initiation. Following non‐processing of the PROSPERO submission, the unchanged protocol was registered with the Open Science Framework (OSF) (https://doi.org/10.17605/OSF.IO/YZR5J). The full protocol together with a detailed account of the registration process is provided in Appendix S1.

### 2.3 Search Strategies and Information Sources

A systematic search of MEDLINE (via PubMed) and EMBASE (via Ovid) covering 2010 to October 2024 was conducted. The search strategy was developed with information specialists. References in included systematic‐review reports were screened for additional relevant studies. Study authors were not contacted. The full line‐by‐line search strategy for each database is provided in Appendix S2.

### 2.4 Eligibility Criteria

#### 2.4.1 Inclusion Criteria

Studies were eligible for inclusion if they met all of the following criteria: (1) The study was a RCT, (2) the study population consisted of individuals diagnosed with knee OA, (3) the study included a placebo control group, (4) at least one active comparator with a presumed direct effect analogous to a medicinal product was included, defined as having a pharmacological, immunological, or metabolic mechanism of action, and (5) pain outcomes were assessed using the WOMAC pain subscale and reported as changes from baseline, including measures of variability (e.g., standard deviation [SD], standard error [SE] or confidence intervals [CI]).

Studies enrolling mixed OA populations (e.g., knee and hip OA) were eligible provided that the study population consisted predominantly of patients with knee OA.

#### 2.4.2 Exclusion Criteria

Studies were excluded if they met any of the following criteria: (1) Publication not in English, Danish, Norwegian, Swedish, or German, (2) studies using food supplements, functional foods, vitamins, or herbal remedies as active comparators, unless the intervention had an established pharmacologically relevant effect and (3) conference abstracts without full‐text publications.

### 2.5 Selection Process

Author J.M.B. conducted the systematic search, exported references, and removed duplicates. Authors J.M.B. and A.R.B. independently reviewed reports from the initial search results according to the eligibility criteria. No articles were translated. Any disagreements were resolved through joint review; persisting disagreements were consulted with a third author (A.B.K.).

All identified studies were screened hierarchically: titles and abstracts first, followed by full‐text review. Reports meeting all inclusion criteria and none of the exclusion criteria were included.

The platform Rayyan was used as a screening management tool (Ouzzani et al. 2016). All reviews and decisions were made manually by the authors without any artificial intelligence assistance.

### 2.6 Data Collection Process and Data Items

Key information was manually extracted from each clinical trial report identified through the search strategy. Three individuals (A.R.B., A.B.K., and J.M.B.) performed the extraction. Each dataset was initially extracted by one author and quality‐controlled by another, ensuring that each author both extracted and reviewed one third of the data.

When data were presented only graphically, values were manually extracted using the online Plotdigitizer application (https://plotdigitizer.com/app).

#### 2.6.1 Data on Trial Characteristics

Data collected included number of study sites, timing of the primary endpoint assessment (if reported), number of clinic visits, total participants and per‐arm allocation, number of treatment arms, study design (parallel group, crossover, etc.), and geographical region(s) and countries. If the primary endpoint timing was not reported, the timing of the latest reported efficacy outcome was used.

#### 2.6.2 Data on Interventional Group Details

Data on interventional groups included the route and frequency of administration, treatment duration, and dose.

#### 2.6.3 Data on Baseline Characteristics

Mean age, sex distribution, proportion of participants of Caucasian race, mean body‐mass index (BMI), distribution of radiographic disease severity (Kellgren–Lawrence [KL] grade), mean WOMAC pain score, and mean Visual Analogue Scale (VAS) pain intensity were collected. The WOMAC pain subscale was selected over function and stiffness because it is the most frequently used outcome.

#### 2.6.4 Data Standardisation

For all reported scales, the range and units were collected to enable standardisation across studies. For continuous variables, all available measures of variability (SD, SEM, CI, IQR, or full range) were recorded and, if not provided as SD, converted accordingly.

### 2.7 Assessment of Methodological Quality in Included Studies

A risk of bias assessment was conducted by one author and checked by another (A.R.B., A.B.K., and J.M.B.), with each author assessing and reviewing one‐third of the reports. The Joanna Briggs Institute Checklist for randomised controlled trials (Barker et al. 2023) was used.

An overall judgement of methodological quality was assigned using a “worst‐item‐counts” approach: studies were rated “low” quality if at least one item was reported as “no”, “unclear” if no items were reported as “no” and at least 1 item was reported as “unclear”, and “high” quality if all items were reported as “yes”.

### 2.8 Assessment of the Certainty of the Body of Evidence

The quality of the body of evidence was evaluated using the GRADE approach (Balshem et al. 2011). This framework uses an overall assessment of evidence based on the risk of bias, small sample bias, inconsistency, imprecision, indirectness, and publication bias.

### 2.9 Estimands and Time‐Points

The primary meta‐analytic outcome was the within‐trial variability of the mean change from baseline in the placebo group for the WOMAC pain subscale. Variability refers specifically to the dispersion of individual patient change scores around the mean within each trial. This measure captures total observed dispersion at the trial level and may reflect a combination of measurement‐related factors, trial design features, and underlying biopsychosocial heterogeneity. The time point was defined as the primary endpoint of the study, or—if not specified—the longest available trial period consistent with the study design. Study windows were pooled, as our outcome of interest was variability, which is often less time‐sensitive than mean change, and pooling increased precision for estimating overall placebo variability.

### 2.10 Data Analysis

All eligible studies were included in the synthesis. Analyses were conducted using R software, version 4.3.2. Descriptive statistics were presented in tables and forest plots.

#### 2.10.1 Standardisation of Outcome Measures

Scores were standardised to a 0–100 scale, and SDs were derived from the reported statistical model's measure of variability, e.g., SE/SEM: SD = SE × √*n*; CI: SD = (CI_upper_—CI_lower_)/(2 × *z*_1–*α*/2_) × √*n*.

Randomisation ratios were standardised as the actual number of participants in the placebo arm divided by the theoretical number under a perfectly balanced design (equal numbers in all arms while maintaining total sample size). A ratio of 1.00 indicates a perfectly balanced design; > 1 indicates relatively more placebo participants; < 1 indicates fewer. Ratios of 1.33 and 0.6 correspond to 2:1 (placebo: active) and 1:2:2 designs, respectively. Thresholds of 0.8 and 1.2 defined unbalanced designs while allowing for minor variation in otherwise balanced studies.

#### 2.10.2 Treatment of Multi‐Report Studies

Three reports were split into multiple studies: Ekman et al. (2014) (studies 1015 and 1018 with different placebo regimens), Conaghan et al. (2013) (three matched placebo arms analysed separately), and Baraf et al. (2010) (two age‐defined subpopulations analysed separately).

#### 2.10.3 Estimation of Variability and Meta‐Analysis

Uncertainty in the primary outcome (variability) was estimated by calculating the sample variance and applying the Chi^2^‐distribution to obtain CIs for the SD. A random‐effects meta‐analysis estimated pooled variability of WOMAC pain change in placebo groups. Heterogeneity was assessed using restricted maximum likelihood estimation and reported as *τ* ^2^ and 95% prediction intervals (PI).

The arithmetic mean is reported as the main outcome for sample‐size calculations. As the primary outcome is a continuous dispersion measure, it is also analysed on a log scale using inverse‐variance weighting to improve assumptions validity and back transformed to report the geometric mean.

#### 2.10.4 Subgroup Analyses

The primary outcome variable was further analysed using linear regression across subgroups and reported as mean (95% CI). No weighting was applied, as variability already accounts for study size (e.g., in SEM or CI).

#### 2.10.5 Sensitivity Analysis

A sensitivity meta‐analysis restricted to studies with high methodological quality was conducted to assess the robustness of the pooled estimates.

#### 2.10.6 Deviations From Protocol

The preregistered protocol specified linear regression analyses to examine contextual factors associated with variability. Additional methodological components including random‐effects meta‐analysis of variability estimates, formal risk‐of‐bias assessment, GRADE evaluation, and prespecified subgroup and sensitivity analyses were incorporated to enhance methodological rigour. These additions did not alter the primary objectives or outcomes of the study.

## 3 Results

### 3.1 Selection of Studies

**FIGURE 1:**
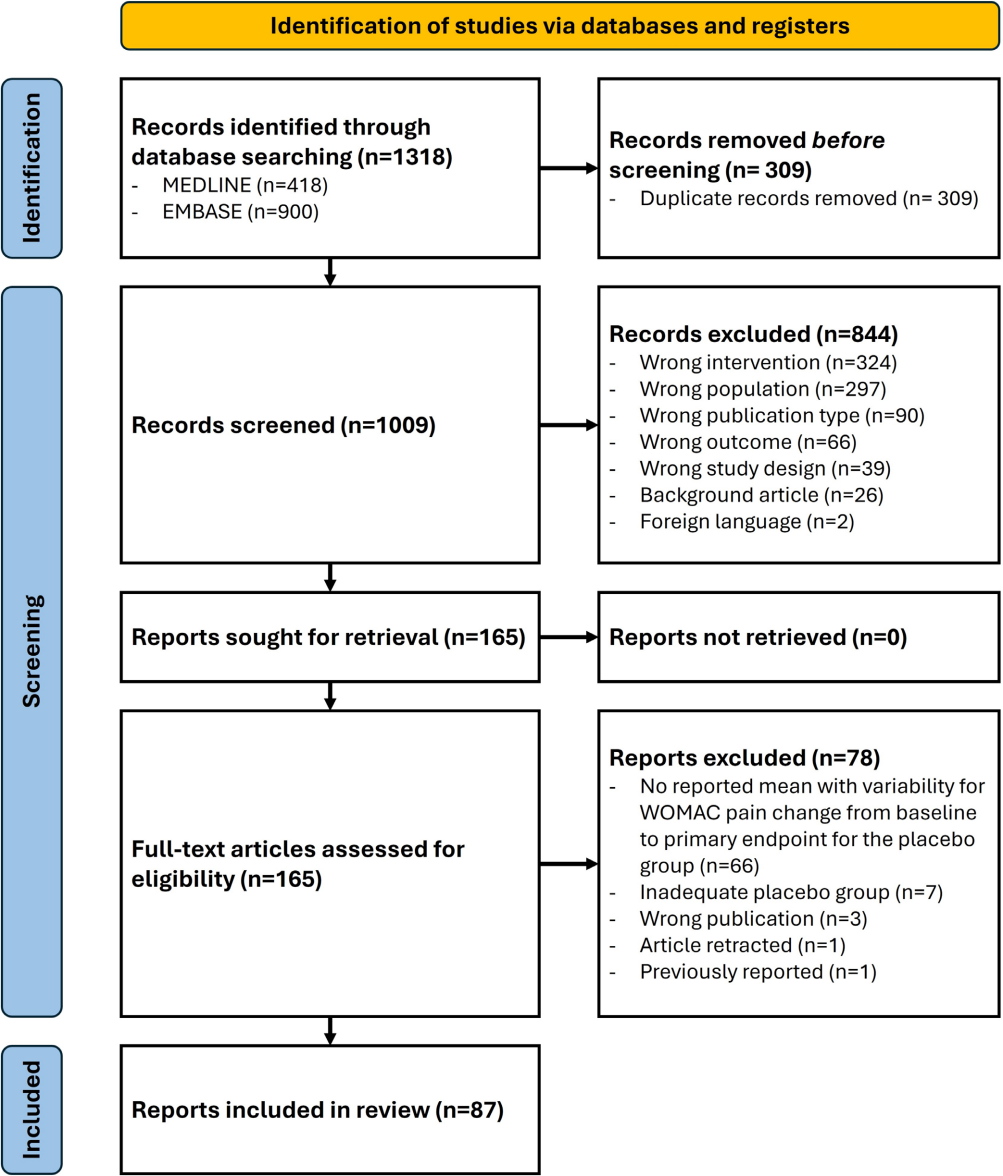
PRISMA flow diagram for systematic reviews.

Of 1318 records, 309 duplicates were removed. A total of 1009 reports were screened by title and abstract. Of these, 165 were eligible for full text review, and 87 reports—including 36,246 participants—were included. Reasons for exclusion are provided in Appendix S3. The search strategy is shown in the PRISMA flowchart in Figure 1.

### 3.2 General Characteristics of Included Studies

Eighty‐seven reports were included (Altman et al. 2015; Arden et al. 2014; Baraf et al. 2010; Berenbaum et al. 2020; Bihlet et al. 2020, 2022; Bihlet, Byrjalsen, Andersen, et al. 2024; Bihlet, Byrjalsen, Mundbjerg, et al. 2024; Birbara et al. 2018; Breivik et al. 2010; Brown et al. 2012; Cai et al. 2019, 2020; Chao et al. 2010; Chevalier et al. 2010; Conaghan et al. 2013, 2020; Conaghan, Cohen, et al. 2018; Conaghan, Hunter, et al. 2018; Dakin et al. 2019; DeCaria et al. 2012; DeLemos et al. 2011; Eckstein et al. 2021; Ekman et al. 2014; Elik et al. 2020; Emadedin et al. 2018; Essex et al. 2012, 2014, 2016; Fleischmann et al. 2019; Frakes et al. 2011; Gibofsky et al. 2014; Gordo et al. 2017; Guehring et al. 2021; Hangody et al. 2018; Hochberg et al. 2019; Huang et al. 2011; Hudson et al. 2021; Hunter et al. 2022; Itoh et al. 2018; Jin et al. 2018; Karsdal et al. 2015; Kelly et al. 2019; Kim et al. 2023; Kosuwon et al. 2010; Krupka et al. 2019; Kuah et al. 2018; Lane et al. 2010, 2024; Langworthy et al. 2019; Lee et al. 2017; Leung et al. 2018; Lohmander et al. 2014; Mayorga et al. 2016; McAlindon et al. 2017b, 2018; Mcmurdo et al. 2016; Nagashima et al. 2011; Nishida et al. 2021a, 2021b; Petterson and Plancher 2019; Pramhas et al. 2023; Prior et al. 2014; Reed et al. 2018; Reginster et al. 2013; Roman‐Blas et al. 2017; Ross et al. 2022; Rovati et al. 2020; Sanga et al. 2017; Schnitzer et al. 2010, 2011, 2019, 2023; Schwappach et al. 2017; Snijders et al. 2011; Spierings et al. 2013; Tiseo et al. 2014; Tive et al. 2019; Treister et al. 2019; Varadi et al. 2013; Veličković et al. 2023; Wang et al. 2021, 2021a, 2021b; Watt et al. 2019; Yazici et al. 2017, 2020), and characteristics are shown in Table 1. Trials had a median (range) of 304 (20, 2980) participants, including 97 (4, 1100) receiving placebo; the median number of study arms was 3 (2, 8), with a median time from baseline to the primary endpoint of 13 (2, 260) weeks, 5 (2, 19) study visits, and 18.5 (1, 129) sites.

**TABLE 1:** Characteristics and overall demographics of trials included in the systematic review.

Trial characteristics	RCTs (*n* = 91)
Trial sample size, *n*, median (min–max)	304 (20–2980)
Placebo group sample size, n, median (min–max)	97 (4–1100)
Publication year, *n* (%)	
2010–2014	32 (35.2)
2015–2019	35 (38.5)
2020–2024	24 (26.4)
Study duration, weeks, median (min–max)	13 (2–260)
Study sites, n, median (min–max)	18.5 (1–129)
Study treatment arms, n, median (min–max)	3 (2–8)
Study visits, n, median (min–max)	5 (2–19)
Joint	
Knee, *n* (%)	75 (82.4)
Hip or knee, *n* (%)	16 (17.6)

Overall trial demographics	Participants (*n* = 36,246)
Mean age, years, median (min–max)	61.3 (45.4–76.8)
Sex, female, *n* (%)	23,536 (65.6)
Race, white, *n* (%)	16,316 (78.0)
Mean BMI, kg/m‑, median (min–max)	30.3 (23.5–33.8)
Mean KL grade, *n* (%)	
KL1	556 (2.4)
KL2	11,223 (48.4)
KL3	9499 (41.0)
KL4	1906 (8.2)
Mean baseline WOMAC pain (0–100), median (min–max)	54.7 (20.8–79.9)
Mean baseline VAS pain (0–100), median (min–max)	63.8 (40.5–75.6)

Placebo participants had a median (range) mean age of 61.3 (54.1, 76.1) years, 66.3 (6.1, 100) percent were female, 83.0 (0, 100) percent were white, a mean BMI of 30.2 (24.8, 33.5) kg/m^2^, and a baseline mean WOMAC pain score of 54.1 (12.0, 80.8) on a normalised 0–100 scale. Most studies focused on knee OA, but some studies included both knee and hip OA (Altman et al. 2015; Berenbaum et al. 2020; Birbara et al. 2018; Breivik et al. 2010; Dakin et al. 2019; DeLemos et al. 2011; Ekman et al. 2014; Gibofsky et al. 2014; Kelly et al. 2019; Lee et al. 2017; Prior et al. 2014; Reed et al. 2018; Sanga et al. 2017; Schnitzer et al. 2019; Spierings et al. 2013; Tive et al. 2019), primarily in trials of drugs targeting nerve growth factor (NGF).

### 3.3 Methodological Quality

Among 87 studies, 50 (57%) were rated as high quality, 12 (14%) were unclear, and the remainder were rated low quality, as presented in Table 2 (McGuinness and Higgins 2021). High‐quality studies included 25,080 (69.2%) participants. The most frequent sources of potential bias were lack of blinding of those administering treatment (*n* = 18, 20%), and differences in care between groups (*n* = 5, 5%). Items most frequently rated as unclear were the use of true randomisation (*n* = 9, 10%) and whether participants were analysed in their randomised groups (*n* = 9, 10%).

**TABLE 2:** Methodological quality assessment.

Study	Q1	Q2	Q3	Q4	Q5	Q6	Q7	Q8	Q9	Q10	Q11	Q12	Q13	Overall
Bihlet, 2024a	High	High	High	High	High	High	High	High	High	High	High	High	High	High
Kang‐Il, 2023	High	High	High	High	High	High	High	High	High	High	High	High	High	High
Bihlet, 2021	High	High	High	High	High	High	High	High	High	High	Low	High	High	Low
Hunter, 2022	High	High	High	High	Low	High	High	High	High	High	High	High	High	Low
Ekcstein, 2021	High	High	High	High	High	High	High	High	High	High	High	High	High	High
Wang, 2021a	High	High	High	High	High	High	High	High	High	High	High	High	High	High
Guehring, 2021	High	High	Low	High	High	High	High	High	High	High	Low	High	High	Low
Nishida, 2021a	High	High	High	High	Low	High	High	High	High	High	High	High	High	Low
Wang, 2021b	High	High	High	High	High	High	High	High	High	High	High	High	High	High
Hudson, 2021	High	High	High	High	High	High	High	High	High	High	High	High	High	High
Bihlet, 2020	High	High	High	High	Low	Low	High	High	High	High	High	High	High	Low
Nishida, 2021b	High	High	High	High	Low	High	High	High	High	High	High	High	High	Low
Yazici, 2020	High	High	High	High	Low	High	High	High	High	High	High	High	High	Low
Berenbaum, 2019	High	High	High	High	High	High	High	High	High	High	High	High	High	High
Conaghan, 2020	High	High	High	High	High	High	High	High	High	High	High	High	High	High
Hochberg, 2019	High	High	High	High	High	High	High	High	High	High	High	High	High	High
Rovati, 2020	High	High	High	High	High	High	High	High	High	High	High	High	High	High
Kelly, 2019	High	High	High	High	Unclear	Low	Unclear	High	High	High	High	High	High	Low
Watt, 2019	High	High	High	High	High	High	High	High	High	High	High	High	High	High
Krupka, 2019	Unclear	High	High	High	Low	High	High	High	High	High	High	High	High	Low
Schnitzer, 2019	High	High	High	High	High	High	High	High	High	High	High	High	High	High
Dakin, 2019	High	High	High	High	High	High	High	High	High	High	High	High	High	High
Elik, 2020	High	High	High	High	Low	Low	Unclear	High	High	Low	High	High	High	Low
Langworthy, 2019	High	High	High	High	Low	High	High	High	High	High	High	High	High	Low
Fleischmann, 2019	Unclear	High	High	High	Unclear	High	Unclear	High	High	High	High	High	High	Unclear
Emadedin, 2018	Unclear	High	High	High	High	High	High	High	High	High	High	High	High	Unclear
Jin, 2018	High	High	High	High	High	High	High	High	High	High	High	High	High	High
Treister, 2019	Unclear	High	High	High	High	High	High	High	High	High	High	High	High	Unclear
Petterson, 2018	Unclear	High	High	High	High	High	High	High	High	High	High	High	High	Unclear
Conaghan, 2018a	High	High	High	High	High	High	High	High	High	High	High	High	High	High
Leung, 2018	High	High	High	High	High	High	High	High	High	High	High	High	High	High
Reed, 2018	High	High	High	High	High	High	High	High	High	High	High	High	High	High
Lee, 2017	High	High	High	High	High	High	High	High	High	Low	High	High	High	Low
Conaghan, 2018b	High	High	Low	High	High	High	High	High	High	High	High	High	High	Low
Yazici, 2017	High	High	Low	High	High	High	High	High	High	High	High	High	High	Low
Hangody, 2018	High	High	High	High	High	High	High	High	High	High	High	High	High	High
Gordo, 2017	High	High	High	High	High	High	High	High	High	High	High	High	High	High
Mayorga, 2016	High	High	High	High	High	High	High	High	High	High	High	High	High	High
Altman, 2015	High	High	High	High	High	High	High	High	High	High	High	High	High	High
McMurdo, 2016	High	High	High	High	High	High	High	High	High	High	Unclear	High	High	Unclear
Essex, 2016	Unclear	High	High	Unclear	High	Unclear	High	High	High	Low	High	High	High	Low
Karsdal, 2014	High	High	High	High	High	High	High	High	High	High	High	High	High	High
Prior, 2014	High	High	High	High	High	High	High	High	High	High	High	High	High	High
Gibofsky, 2014	High	High	High	High	High	High	High	High	High	High	High	High	High	High
Lohmander, 2014	High	High	High	High	High	High	High	High	High	High	High	High	High	High
Tiseo, 2014	High	High	High	High	High	High	High	High	High	High	Unclear	High	High	Unclear
Varadi, 2013	High	High	Unclear	High	High	High	High	High	High	High	Unclear	High	High	Unclear
Arden, 2013	High	High	High	High	Low	High	High	High	High	High	High	High	High	Low
Spierings, 2012	High	High	High	High	High	High	High	High	High	High	High	High	High	High
Essex, 2012	High	High	High	High	High	High	High	High	High	High	Unclear	Low	High	Low
Reginster, 2012	High	High	High	High	High	High	High	High	High	High	High	High	High	High
Brown, 2012	Unclear	High	High	High	High	High	High	High	High	High	High	High	High	Unclear
DeCaria, 2011	High	High	High	High	High	High	High	High	High	High	High	High	High	High
Nagashima, 2011	Unclear	High	High	High	Unclear	High	High	High	High	High	High	High	High	Unclear
Huang, 2013	Unclear	High	High	High	Unclear	High	Unclear	High	High	High	High	High	High	Unclear
Snijders, 2011	High	High	High	High	High	High	High	High	High	High	High	High	High	High
Kosuwon, 2010	High	High	High	High	High	High	High	High	High	High	Unclear	High	High	Unclear
Lane, 2010	High	High	High	High	High	High	High	High	High	High	High	High	High	High
Schnitzer, 2011	High	High	High	High	High	High	High	High	High	High	High	High	High	High
DeLemos, 2011	High	High	High	High	High	High	High	High	High	High	High	High	High	High
Schnitzer, 2010	High	High	High	High	High	High	High	High	High	High	High	High	High	High
Chao, 2010	High	High	High	High	Low	High	High	High	High	Low	Unclear	High	High	Low
Chevalier, 2010	High	High	High	High	Low	High	High	High	High	High	High	High	High	Low
Lane, 2024	High	High	High	High	High	High	High	High	High	High	High	High	High	High
Bihlet, 2024b	High	High	High	High	Low	High	High	High	High	High	High	High	High	Low
Velickovic, 2023	High	High	High	High	High	High	High	High	High	High	Unclear	High	High	Unclear
Pramhas, 2023	High	High	High	High	High	High	High	High	High	High	High	High	High	High
Schnitzer, 2023	High	High	High	High	High	High	High	High	High	High	High	High	High	High
Ross, 2022	High	High	High	High	Low	High	High	High	High	High	High	High	High	Low
Wang, 2021c	High	High	High	High	High	High	High	High	High	High	High	High	High	High
Cai, 2020	High	High	High	High	High	High	High	High	High	High	High	High	High	High
Tive, 2019	High	High	High	High	High	High	High	High	High	High	High	High	High	High
Cai, 2019	High	High	High	High	High	High	High	High	High	High	High	High	High	High
McAlindon, 2018	High	High	High	High	High	High	High	High	High	High	High	High	High	High
Kuah, 2018	High	High	High	High	Low	High	High	High	High	High	High	High	High	Low
Birbara, 2018	High	High	High	High	High	High	High	High	High	High	High	High	High	High
Itoh, 2018	High	High	High	High	High	High	High	High	High	High	High	High	High	High
McAlindon, 2017	High	High	High	High	Low	High	High	High	High	High	High	High	High	Low
Sanga, 2017	High	High	High	High	High	High	High	High	High	High	High	High	High	High
Roman‐Blas, 2017	High	High	High	High	High	High	High	High	High	High	High	High	High	High
Schwappach, 2017	High	High	High	High	High	High	High	High	High	High	High	High	High	High
Essex, 2014	High	High	High	High	High	High	High	High	High	High	High	High	High	High
Frakes, 2011	High	High	High	High	High	High	High	High	High	High	High	High	High	High
Breivik, 2010	High	High	High	High	High	High	High	High	High	High	High	High	High	High
Ekman, 2014	High	High	High	High	High	Low	High	High	High	High	Unclear	High	High	Low
Conaghan, 2013	High	High	High	High	Low	High	High	High	High	High	High	High	High	Low
Baraf HSB, 2011	High	High	High	High	High	High	High	High	High	High	High	High	High	High

### 3.4 Variability of WOMAC Pain Change From Baseline to Primary Endpoint in the Placebo Group

**FIGURE 2:**
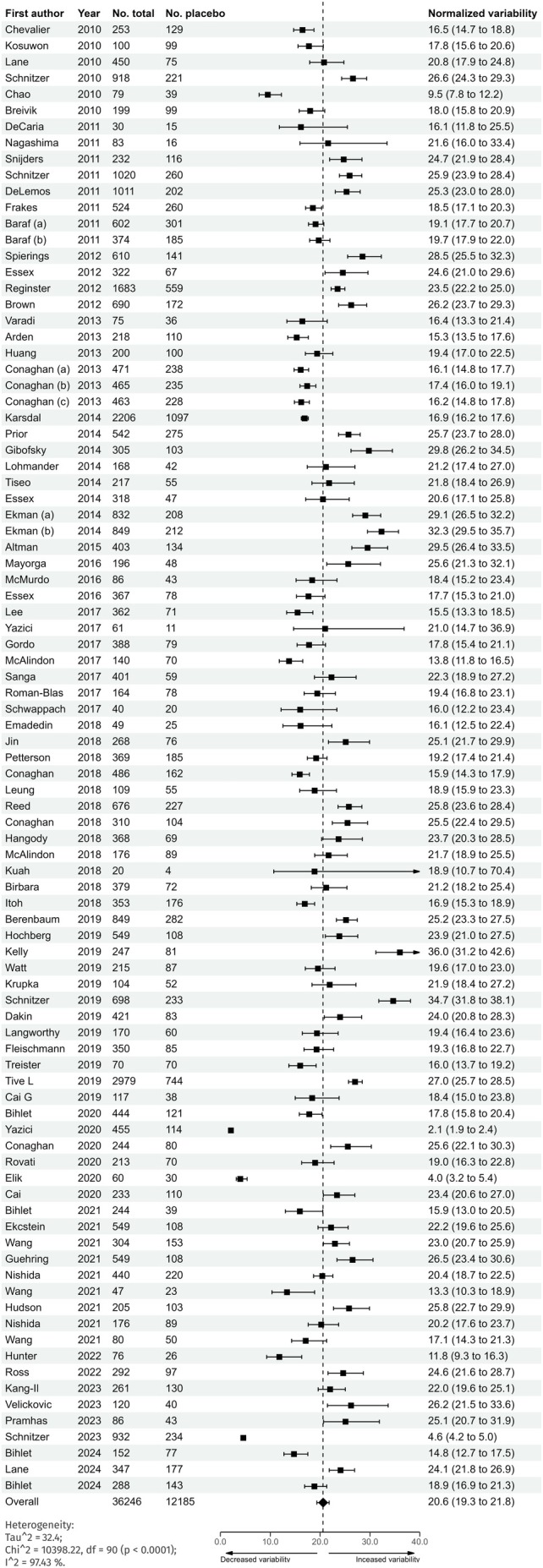
Normalised variability of WOMAC pain intensity subscale changes from baseline to primary endpoint in the placebo group with 95% confidence intervals.

The arithmetic mean variability (SD) of WOMAC pain change across studies was 20.6 (95% CI: 19.3–21.8) on a normalised estimated scale of 0–100 in the random‐effects meta‐analysis, as shown in Figure 2. Variability was substantial (*τ* ^2^ = 32.4, 95% prediction interval: 9.3–31.8), indicating that differences occurred primarily between studies. The geometric mean variability (SD) was 19.55 (95% CI: 18.0–21.3; *τ* ^2^ = 0.16 referring to log[SD]).

No significant correlation was observed between variability and the number of placebo participants (Pearson *r* = 0.13), indicating that study size alone did not account for the observed differences.

### 3.5 Trial Characteristics

**FIGURE 3:**
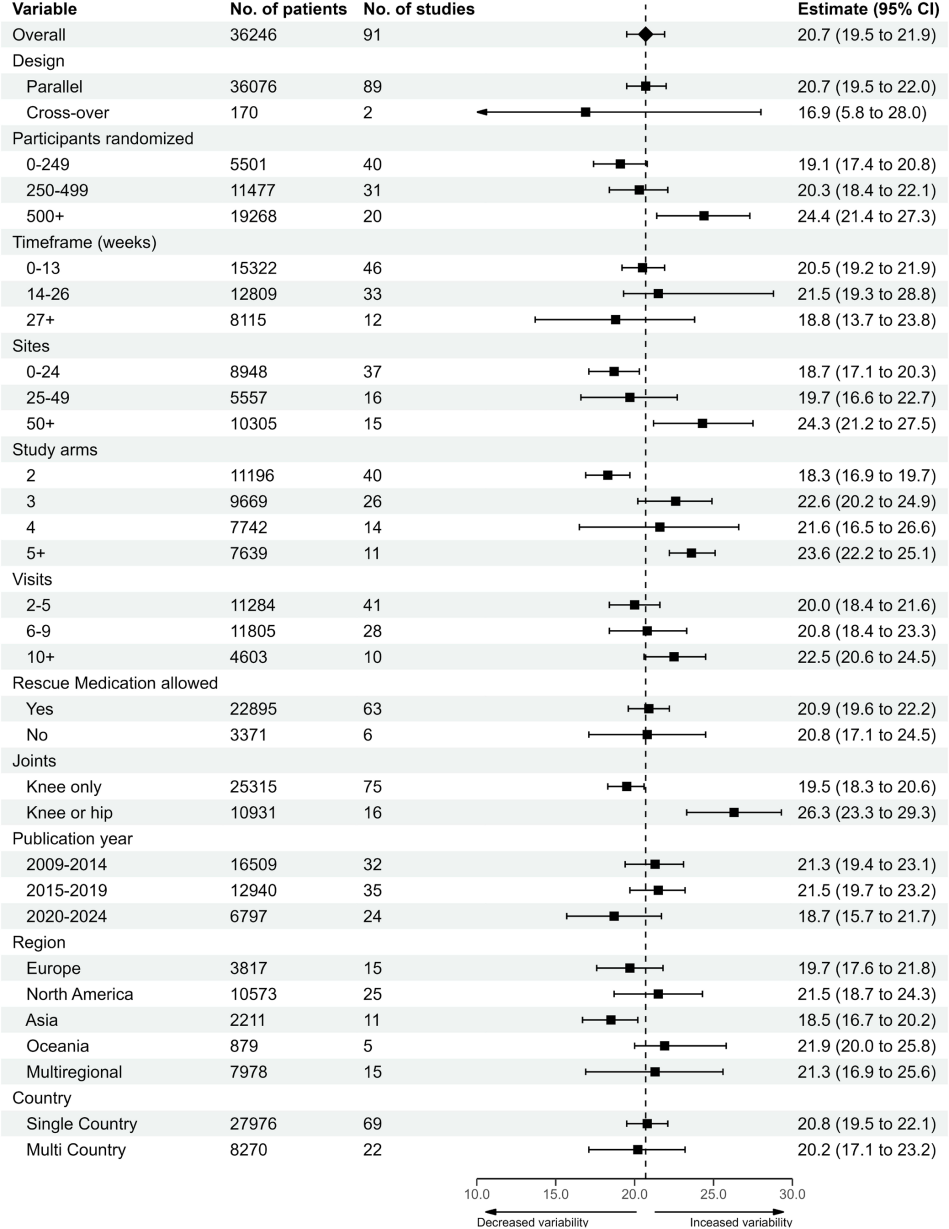
The association of trial characteristics with variability of WOMAC pain change from baseline to primary endpoint in the placebo group.

Figure 3 presents the association between trial characteristics and variability in WOMAC pain change. Smaller trials had lower variability (0–249 participants: 19.1 [95% CI: 17.4–20.8]), whereas larger trials had higher variability (250–499: 20.3 [95% CI: 18.4–22.1]; 500+: 24.4 [95% CI: 21.4–27.3]). Trials with fewer study sites had lower variability (0–24 sites: 18.7 [95% CI: 17.1–20.3]), whereas trials with more sites were associated with increased variability (25–49: 19.7 [95% CI: 16.6–22.7]; 50+: 24.3 [95% CI: 21.2–27.5]).

Study duration did not affect variability, although trials with more study visits demonstrated slightly higher variability (2–5 visits: 20.0 [95% CI: 18.4–21.6]; 6–9: 20.8 [95% CI: 18.4–23.3]; 10+: 22.5 [95% CI: 20.6–24.5]). Trials with more study arms were similarly associated with increased variability (2 arms: 18.3 [95% CI: 16.9–19.7]; 3: 22.6 [95% CI: 20.2–24.9]; 4: 21.6 [95% CI: 16.5–26.6]; 5+: 23.6 [95% CI: 22.2–25.1]).

Trials including both hip and knee OA showed higher variability (26.3 [95% CI: 23.3–29.3]) than trials including knee OA only (19.5 [95% CI: 18.3–20.6]). Rescue medication use, geographical region, and single‐ versus multi‐country conduct had minimal impact. Only two cross‐over studies were included, resulting in a wide CI of the aggregate estimate, and the results should be interpreted with caution. More recent studies tended to have a slightly lower variability.

In summary, trials with greater numbers of participants, sites, or study arms, and those including both hip and knee OA were associated with higher variability.

### 3.6 Placebo Comparator Group Characteristics

**FIGURE 4:**
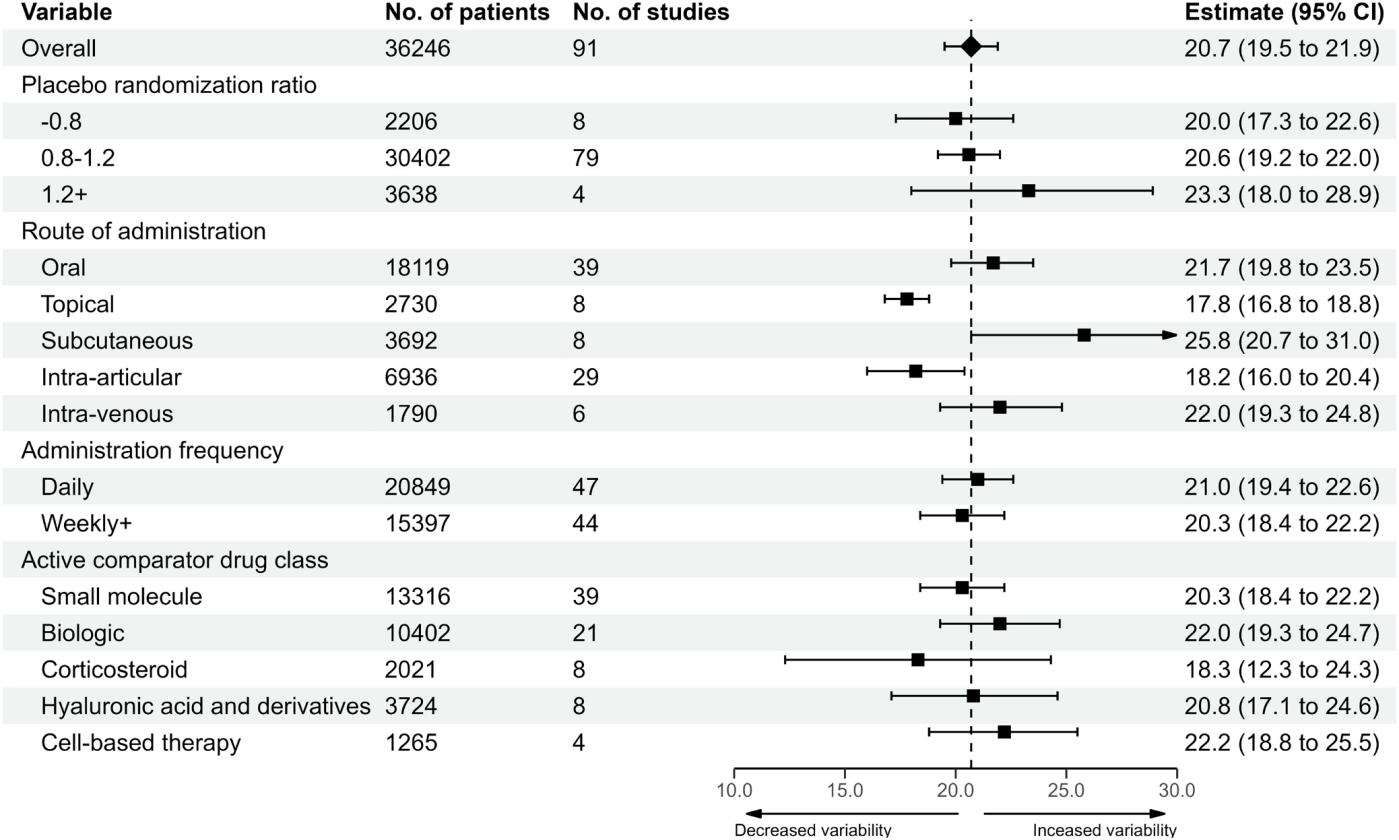
The association of placebo drug characteristics with variability of WOMAC pain change from baseline to primary endpoint in the placebo group.

The influence of placebo drug characteristics on variability is illustrated in Figure 4. Topical and intra‐articular placebos were associated with lower variability than oral administration (oral: 21.7 [95% CI: 19.8–23.5]; topical: 17.8 [95% CI: 16.8–18.8]; intra‐articular: 18.2 [95% CI: 16.0–20.4]). Randomisation ratio, administration frequency, and active comparator class were associated with minimal impact. Pooling across administration routes assumed transitivity for variability. These exploratory analyses were not intended to imply causality, as comparator selection is primarily driven by efficacy, safety, and practical considerations.

### 3.7 Baseline Clinical Characteristics of the Placebo Group

**FIGURE 5:**
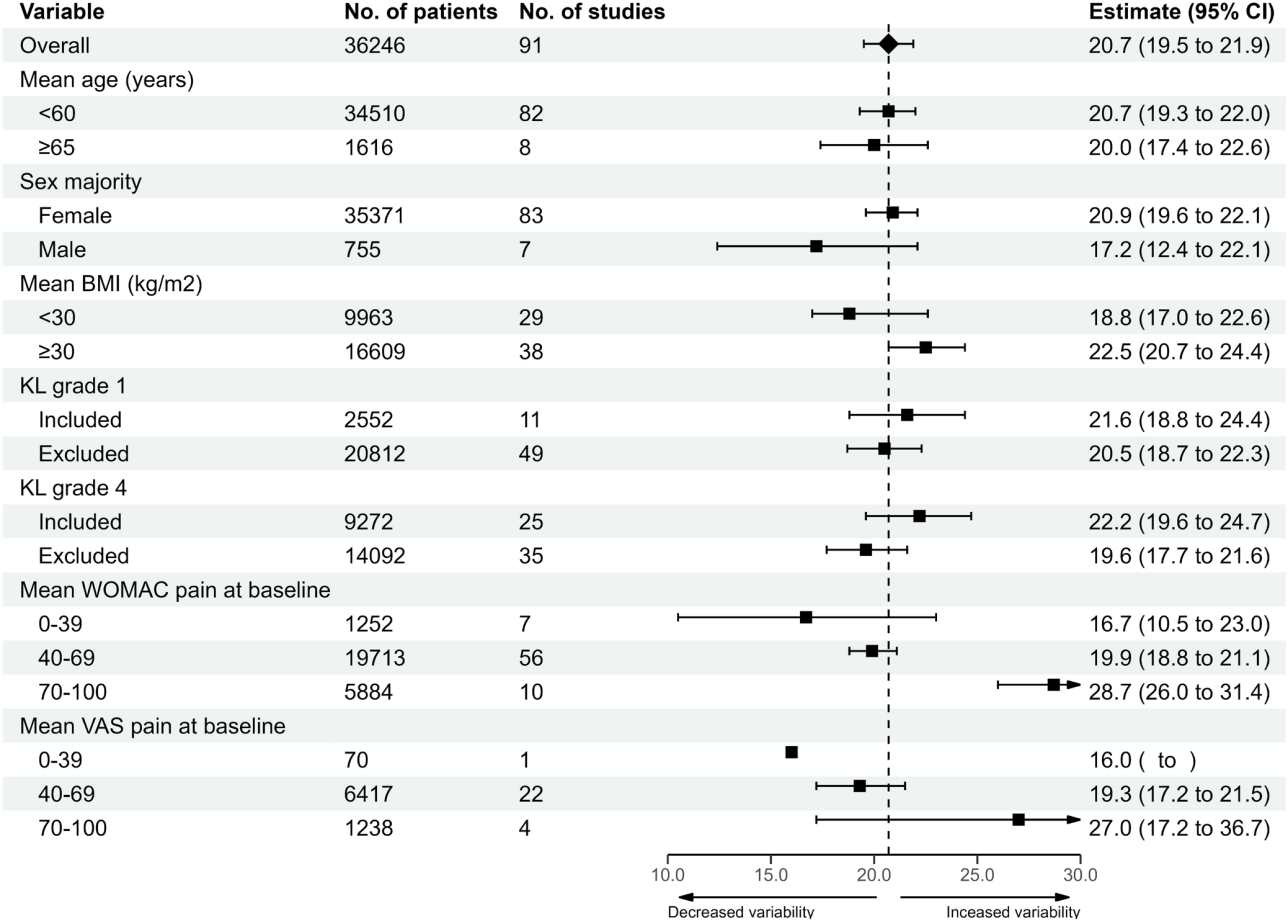
The association of placebo group baseline characteristics with variability of WOMAC pain change from baseline to primary endpoint in the placebo group.

The association of baseline characteristics with variability is illustrated in Figure 5. Trials with higher mean BMI were associated with greater variability (< 30: 18.8 [95% CI: 17.7–22.6]; ≥ 30: 22.5 [95% CI: 20.7–24.4]). Age and sex had minimal impact, although few studies included most male participants. The inclusion of KL grade 1 and/or 4 participants did not affect variability. Higher mean baseline pain scores (WOMAC or VAS) were associated with greater variability at the primary endpoint (WOMAC 0–39: 16.7 [95% CI: 10.5–23.0, single‐study estimate]; 40–69: 19.9 [95% CI: 18.8–21.1]; 70–100: 28.7 [95% CI: 26.0–31.4]).

### 3.8 Sensitivity Analyses

A sensitivity meta‐analysis restricted to studies with high methodological quality gave a slightly higher mean variability (SD) in WOMAC pain change than observed in the primary analysis, with an arithmetic mean of 21.8 (95% CI: 20.4–23.2) on the normalised 0–100 scale. Between‐study heterogeneity remained notable (*τ* ^2^ = 23.2), and the 95% PI ranged from 12.3 to 31.6.

### 3.9 Certainty of the Evidence

The certainty of the evidence for variability (SD) in WOMAC pain change from baseline was rated as moderate, see Table 3.

**TABLE 3:** Certainty of the evidence.

Domain	Risk of bias	Small sample bias	Inconsistency	Imprecision	Indirectness	Publication bias	Certainty
Variability (SD) in WOMAC pain change from baseline	Do not downgrade	Do not downgrade	Downgrade	Do not downgrade	Do not downgrade	Do not downgrade	Moderate

## 4 Discussion

This systematic review is the first to quantify variability in longitudinal pain reporting in OA RCTs, specifically examining normalised SD estimates derived from each study's reported measure of variability. The focus was on the WOMAC pain intensity subscale score change from baseline to the primary endpoint in placebo groups. The analysis included 87 reports with a total of 36,246 study participants. This measure captures total observed variability and may reflect a combination of measurement‐related factors, trial design features, and underlying mechanistic and phenotypic heterogeneity within the study population.

The results provide an indicative overview of the expected variability of this key outcome in OA trials, acknowledging the wide prediction interval, and identify trial‐level factors associated with variability. Key factors included trial design characteristics, placebo administration route, and trial‐level participant clinical characteristics. While cautious inferences can be made about the influence of individual parameters, comparisons across parameters may not be meaningful. Consequently, this review does not rank individual parameters by their impact.

### 4.1 Implications for Clinical Trials

Variability in key outcome assessments has significant implications for OA trial design. Higher variability reduces statistical power, necessitating larger sample sizes to detect meaningful differences and increasing trial costs and complexity. It can obscure treatment effects, potentially leading to false‐negative results. This is particularly relevant in the development of new OA therapies, where high placebo responses (PRs) are believed to have contributed to the long list of previous OA trial failures (Huang et al. 2019; Karsdal et al. 2016).

For example, assuming a minimal clinically important difference for WOMAC pain in knee OA of 12 on a 0–100 scale (Angst et al. 2018; Conaghan et al. 2022; Ehrich et al. 2000; MacKay et al. 2019; Silva et al. 2023; Tubach et al. 2005), required sample sizes vary substantially depending on variability (*n* = 116 for SD = 22.7; *n* = 286 for SD = 36.0; power = 0.8, alpha = 0.05, independent two‐sample *t*‐test). Using trial‐level results and multivariate linear regression adjusted for baseline WOMAC pain, we can predict sample size effects of modifiable characteristics: assuming Δ12 and SD = 20, adding 10 study sites requires additionally 1.4 (95% CI: −0.4–3.3) participants per arm (3.2% increase), whereas adding an extra study arm requires additionally 5.0 (95% CI: 0.8–9.5) participants per arm (11.3% increase), in addition to the added arm.

Although differences in variability across study population characteristics are relevant for trial planning, some variability is inevitable when using patient‐reported outcomes and excluding large participant groups to reduce variability may not be feasible. Importantly, some sources of variability may represent true mechanistic and biopsychosocial heterogeneity rather than measurement noise or trial logistics. Future studies could address this by incorporating baseline mechanistic phenotyping, pre‐specified stratified or subgroup analyses by phenotype, or multilevel models to partition variance attributable to participant‐level, site‐level, and measurement‐level factors to help distinguish stable patient‐level heterogeneity from avoidable sources of variability. Recognising these contributions ensures that strategies to reduce variability do not inadvertently exclude phenotypically diverse participants.

Over time, RCTs for chronic pain have shown consistent baseline pain levels and drug responses but increasing PRs that reduce treatment benefit (Katz et al. 2008; Tuttle et al. 2015). Interestingly, our results indicate a recent decreasing trend in PR variability, which may reflect improvements in OA trial methodology and may partially counteract reductions in active‐placebo differences.

### 4.2 Trial Design Characteristics and Variability

Smaller studies and those with fewer sites demonstrated lower variability in WOMAC pain. This is consistent with meta‐epidemiological evidence showing that trials with smaller sample sizes (Dechartres et al. 2013) and single‐centre designs (Dechartres et al. 2011) tend to report larger estimated treatment effects. While smaller, single‐site studies may reduce variability and logistical complexity, sufficient sample size and multiple sites are often necessary for statistical power and recruitment. Fewer sites may limit geographical and demographic diversity, potentially reducing generalisability and raising concerns for regulatory review (Rothwell 2005; FDA CDER 2025).

Study design also influenced variability: two‐arm trials showed lower variability than multi‐arm trials, suggesting that reduced complexity can improve focus on primary comparisons. Study duration did not impact variability, suggesting that longer follow‐up does not necessarily introduce additional noise. The wide range of study durations illustrates the heterogeneity of OA trial designs, while the absence of a duration effect supports pooling variability across different temporal contexts while acknowledging study design heterogeneity. By contrast, a higher number of study visits was associated with slightly increased variability, potentially reflecting measurement error or participant fatigue. These factors should be considered when designing visit schedules.

Some included trials enrolled mixed populations of patients with knee and hip OA, although knee OA is predominant. Trials including both hip and knee OA populations showed higher variability than knee‐only trials. However, the contribution of hip OA participants to this variability cannot be determined from trial‐level data, and individual participant data (IPD) analyses would be required to clarify joint‐specific effects.

### 4.3 Drug and Clinical Characteristics and Variability

Placebo administration route influenced variability, with topical and intra‐articular placebo associated with lower variability. Route of administration has previously been found an important modifier of PR in OA (Bannuru, McAlindon, et al. 2015; Zhang et al. 2008). Other factors, including randomisation ratio and administration frequency, did not significantly impact variability, suggesting these factors are less critical in controlling variability.

### 4.4 Placebo Response in OA Trials

PR is a key determinant of study power in OA and pain research. Factors associated with variability in this review align with prior studies examining trial characteristics associated with increased PR (Abhishek and Doherty 2013; Doherty and Dieppe 2009; Dworkin et al. 2011; Irizarry et al. 2009; Katz et al. 2008; Tuttle et al. 2015; Vase 2019; Vase et al. 2015; Wen et al. 2022; Zhang et al. 2008; Zou et al. 2016). The impact of clinical characteristics on PR has been studied with mixed results (Häuser et al. 2011; Vase 2019). Although stratifying participants by these factors may theoretically reduce variability, such strategies may increase screen failure rates and recruitment challenges, and their success has been limited (Dworkin et al. 2011; Farrar et al. 2014; Vase 2019).

### 4.5 Limitations and Strengths of This Study

This review uses meticulous methodology to analyse a wide range of RCTs, providing an overview of variability in pain reporting and its implications for OA trial design. Understanding factors contributing to variability may optimise trial efficiency and cost‐effectiveness.

Several limitations must be acknowledged. Included trials varied in sample sizes, demographics, interventions, follow‐up durations, and calculation methods, introducing heterogeneity. Study‐level data may be subject to aggregation bias, and IPD could improve precision and clarify whether variability is driven by participant composition or trial procedures, although the estimand was clearly stated, and random‐effects meta‐regression model between‐study variation. Sensitivity analyses were consistent with the primary meta‐analysis, supporting the robustness of results. Limited availability of data for certain factors restricts generalisability. Wide ranges in certain variability estimates reduce confidence and should be considered during interpretation. Publication bias is possible, as grey literature was not searched, although symmetrical funnel plots suggest minimal bias (Appendix S4), with a single small, high–risk‐of‐bias study with a large standard error located near the pooled estimate.

While trial‐level analyses limit the ability to distinguish mechanistic sources of variability, acknowledging that variability in patient‐reported outcome may reflect meaningful biopsychosocial heterogeneity complements the statistical perspective. Recognising this perspective highlights that variability may represent underlying patient diversity rather than solely methodological noise.

This analysis focused on pain outcomes. Other important OA outcomes, such as functional disability, were outside the scope and warrant further investigation.

### 4.6 Future Directions

Focusing on placebo participants enhances comparability across studies by minimising confounding from active treatments and allowing intrinsic contributors to variability to be examined. Future research should explore the impact of these factors on the treatment difference between active and placebo groups and overall assay sensitivity, which may vary depending on whether the variability is affected equally or differently across groups. IPD meta‐analyses could further clarify the contribution of trial characteristics, drug attributes, and participant factors, thereby improving the design and reliability of OA trials and facilitating development of effective therapies.

## 5 Conclusion

This systematic review demonstrates substantial variability in pain reporting across OA RCTs and identifies key factors associated with placebo‐arm variability. Larger trials (more participants, sites, arms, and visits) and trials with higher baseline pain were associated with greater variability, whereas trials with topical and intra‐articular interventions showed lower variability. These findings indicate that common design choices and participant characteristics influence trial variability and should be considered in study planning. Although this analysis focused on placebo‐arm variability, between‐group variability is critical for trial efficiency and assay sensitivity. These variability estimates may inform sample‐size calculations for enhanced trial precision and support the development of effective OA treatments, although substantial heterogeneity should be considered.

## Author Contributions

Conception and design: J.M.B., A.R.B. Literature search: J.M.B. Study selection: J.M.B., A.R.B. Data extraction: J.M.B., A.B.K., A.R.B. Analysis and interpretation of the data: J.M.B., A.B.K., L.A.‐N., A.R.B. Drafting of the article: J.M.B. Critical revision of the article for important intellectual content: J.M.B., A.B.K., L.A.‐N., A.R.B. Final approval of the article: J.M.B., A.B.K., L.A.‐N., A.R.B.

## Funding

No specific funding was received for this study. All authors were compensated through their regular salaries, and the funding source had no role in the study design, data collection, analysis, interpretation, or writing of the manuscript.

## Disclosure

*Use of Artificial Intelligence*: No AI tools were used in the preparation of this manuscript.

The Patient and Public Involvement Statement: Patients or members of the public were not involved in the design of the trial.

## Ethics Statement

The authors have nothing to report.

## Conflicts of Interest

The authors declare no conflicts of interest.

## Supporting information


**Appendix S1:** Research protocol.
**Appendix S2:** Search strategy and search protocol.
**Appendix S3:** Exclusions with reason after full‐text screening.
**Appendix S4:** Funnel plot.

## Data Availability

The data are publicly available.
