# Supplementary material for: Factors Contributing to Variability in Longitudinal Pain Scores in Osteoarthritis Randomised Clinical Trials: A Systematic Review and Meta‐Analysis
Source: Eur J Pain. 2026 May 11;30:e70283. doi: 10.1002/ejp.70283 (PMC13161740; doi:10.1002/ejp.70283)
Supplement: Supplementary file 1 — Appendix S1: Research protocol. Appendix S2: Search strategy and search protocol. Appendix S3: Exclusions with reason after full‐text screening. Appendix S4: Funnel plot. [file EJP-30-0-s001.docx]

**Appendices:**

Appendix 1: Research protocol

- Page: 2

Appendix 2: Search strategy and search protocol

- Page: 11

Appendix 3: Exclusions with reason after full-text screening:

- Page: 14

Appendix 4: Funnel plot

- Page: 25

Appendix 1:

Research Protocol

**Evaluating variability in knee osteoarthritis trials through a comparative analysis of standard deviations in WOMAC pain scores**

**Protocol for a systematic review of randomised controlled trials**

**Asger Reinstrup Bihlet^1^, Andrea Bak Kaaber^1^, Jakob Mejdahl Bentin^1,2^**

1 – NBCD A/S

2 – Aalborg University, Center for Neuroplasticity and Pain (CNAP)

**Corresponding author:**

Asger Reinstrup Bihlet, MSc, PhD

E-mail: abi@nbcd.com

NBCD A/S

Telefonvej 8d, 2860 Søborg

Denmark

**Registration:** The protocol has been submitted for registration at PROSPERO: 26NOV2024 (#619082).

**Keywords:** Systematic review, randomised controlled trials, knee osteoarthritis, placebo, pain, treatment, standard deviation, variability, WOMAC

Summary

Objective

The objective of the study is to compare pain assessment variability in knee osteoarthritis randomised controlled trials. Insights into pain assessment variability may help improve trial assay sensitivity.

Methods

Study design

This is a systematic review and meta-analysis of placebo-controlled randomised trials for at least one pharmacological intervention in people with knee osteoarthritis.

Outcomes of interest

We will consider the primary efficacy outcome domain to be patient-reported pain per default, which must be reported at least using the Western Ontario and McMaster Universities Arthritis Index (WOMAC) pain subscale including data describing data variability. We will collect trial characteristics, intervention variables, contextual factors, and other common pain assessment measures.

Sampling methods

MEDLINE and EMBASE will be searched from 2010 to present. Two reviewers will select the eligible papers based on in- and exclusion criteria and assess study characteristics.

Statistical analyses

Data will be standardised, tabulated and illustrated.

Expected results

We expect a standard deviation of pain efficacy measurement using WOMAC pain in knee osteoarthritis RCTs in the range of 15-40 on a normalised scale of 0-100.

We expect to be able to describe commonly reported RCT characteristics associated with a higher or lower standard deviation in the main outcome parameters or pain.

Discussion

The main topic of discussion is the potential impact of the observed differences in standard deviations in OA clinical trials, and other observations in terms of how variability may relate to commonly reported trial and population characteristics.

Background

Literature summary

Assay sensitivity (AS) in randomised clinical trials (RCTs) refers to the ability to detect differences between a treatment and a control, with a lack of AS potentially leading to false conclusions about (lack of) treatment efficacy. This is particularly important in conditions like osteoarthritis (OA), a widespread and disabling joint disease characterised by pain, where there is a pressing need for new treatments. However, the subjective nature of pain and reliance on patient-reported outcome measures (PROMs) introduces variability in trial results, complicating the assessment of treatment effectiveness. Efforts to improve AS focus on either increasing the difference in outcomes between treatment and control or reducing variability without affecting the effect size. This study aims to evaluate the variability in pain assessment across OA trials to better understand AS.

Justification

An analysis of RCTs from 1990 to 2013 revealed stable baseline pain and stable drug responses but increased placebo responses (PRs), reducing the observed treatment advantage [1]. The size of the PR is the most crucial factor influencing the outcome of pain trials [2], and large PRs in OA trials are thought to have played a role in the numerous trial failures to date [3–5]. Investigations of PRs focus mostly on differences in treatment effect, while more studies of variability of treatment effect are needed. In OA clinical trials pain is widely assessed with the extensively validated Western Ontario and McMaster Universities Arthritis Index (WOMAC) [6].

Relevance

OA is the most prevalent joint disease globally, impacting about 10% of men and 18% of women over 60 [7], yet it has few safe and effective treatment options [8,9]. Given the high prevalence, the disability associated with OA, and the lack of effective therapies that can reverse or delay the disease, it is crucial to explore and develop new treatment options. Insights into treatment effect variability might make it possible to improve AS to the benefit of patients and practitioners.

Feasibility

Given our prominent position in the field, our deep insights into the existing literature, and our academic expertise, we can conduct this systematic review.

Purpose, Objectives and Hypothesis

Purpose of the study

The purpose of the study is to gain insights into OA pain assessment variability to improve AS.

Objectives

The review will address questions related to patients with knee OA who have participated in placebo-controlled RCTs of pharmacological interventions where pain was assessed using the WOMAC pain subscale.

Primary:

- Compare pain assessment variability in OA RCT placebo groups in efficacy domain WOMAC pain subscale.

Secondary:

- Compare pain assessment variability in OA RCTs in efficacy domains Numeric Pain Rating Scale (NRS), Visual Analogue Scale (VAS), weekly average of daily pain, Knee injury and Osteoarthritis Outcome Score (KOOS), Intermittent and Constant Osteoarthritis Pain (ICOAP), and Patient Global Assessment (PGA).
- Estimate the association between participant baseline characteristics, study characteristics (e.g. number of participants and number of clinical centres involved) and pain assessment variability.

Hypotheses

- There are substantial differences in pain assessment variability across RCTs.
- Participant baseline characteristics are not associated with pain assessment variability.
- Trial characteristics previously found to be associated with a larger placebo treatment effect (section 5.6.3) are also associated with a larger pain assessment variability.

Populations

The study population will be defined by the included trials. The target population will be adults with symptomatic knee osteoarthritis.

Methods

The protocol follows the PRISMA-P guideline for protocol reporting [10].

Protocol and registration

The protocol has been submitted for registration at PROSPERO: 26NOV2024 (#619082).

Eligibility criteria

Inclusion criteria

1. Randomised controlled trial.
2. Study population is people with knee OA.
3. Trials including a placebo group.
4. At least one active comparator with an assumed direct effect compatible with that of a medicinal product, ie either a pharmacological, immunological or metabolic effect.
5. Pain assessment with WOMAC pain subscale including variability.

Exclusion criteria

1. Publications in other languages than English, Danish, Norwegian, Swedish, or German.
2. Food supplements, functional foods, vitamins, or herbal remedies with no established pharmacologically relevant effect as active comparator.
3. Conference abstracts.

Information sources

A systematic search of MEDLINE (via PubMed) and EMBASE (via Ovid) will be searched. Databases will be searched from 2010 until present. Eligible studies will also be found by manually screening the reference lists of systematic literature reviews or meta-analyses from the search, as well as the reference lists of included studies.

Search strategy

PubMed

(((((("Osteoarthritis, Knee"[Mesh]) OR (knee osteoarthritis[Text Word])) OR (osteoarthritis of the knee[Text Word])) OR ("Musculoskeletal Pain"[Mesh])) AND ((("Placebos"[Mesh]) OR ("Placebo Effect"[Mesh])) OR (placebo*[Text Word]))) AND ((("Western Ontario and McMaster Universities Arthritis Index"[Text Word]) OR (WOMAC[Text Word])) OR ("Pain Measurement"[Mesh]))) AND ("Randomized Controlled Trial" [Publication Type]) Filters: from 2010 - 2024

Embase

('knee osteoarthritis'/exp OR 'knee osteoarthritis':ti,ab,kw OR 'osteoarthritis of the knee':ti,ab,kw OR 'musculoskeletal pain'/exp) AND ('placebo'/exp OR 'placebo effect'/exp OR placebo*:ti,ab,kw) AND ('pain measurement'/exp OR 'western ontario and mcmaster universities osteoarthritis index':ti,ab,kw OR womac:ti,ab,kw) AND 'randomized controlled trial'/exp AND [2010-2024]/py

Study records

Data management

All references will be imported to Rayyan [11]. Data will be extracted manually and plotted into a customised database, where it will be stored.

Selection process

Author JB will perform the systematic search, reference export, and duplicate removal. Authors JB and AB will independently sort the remaining references based on eligibility criteria and screen reference lists. Potential disagreements will be solved by discussion, and potential persisting disagreements will be consulted with a third reviewer (AK).

All identified studies will be screened for eligibility in a hierarchical manner:

1. Screening of title and abstract.
2. Full text screening.

References fulfilling all inclusion criteria, and no exclusion criteria are considered eligible and included in meta-analysis.

Data collection process

Authors AB and JB will manually extract data from references to the customised database. The preferred time point for outcome will be the time point described by the study as the primary endpoint; if no time point is pre-specified as primary endpoint, the longest possible trial period (respecting the original trial design) will be extracted.

Reference authors will not be contacted during the process.

Data items

All identified references:

The following items will be attempted to be collected on all identified references to the extent possible:

- Inclusion (with an identification number) or exclusion (with the reason for exclusion).

Background

- First author.
- Year of publication.
- Journal of publication.

Trial characteristics

- Study design.
- Number of participants randomised in total.
- Timeframe from baseline to primary endpoint (weeks).
- Number of sites.
- Number of study arms.
- Number of planned face-to-face visits from baseline to primary endpoint (weeks).

Intervention

For placebo arm(s):

- Number of participants.
- Route of administration.
- Frequency of administration.
- Treatment duration.

For pharmacological intervention arm(s):

- Number of participants.
- Generic name.
- Route of administration.
- Dose.
- Unit.
- Frequency.
- Treatment duration.

Contextual factors

- Age (years), preferably mean.
- Sex (percentage women).
- BMI (kg/m^2^), preferably mean.
- Kellgren-Lawrence radiological grade.
- Race (percentage white).

Efficacy measures

- Baseline and primary endpoint timeframe WOMAC pain subscale score and variability.
- Baseline and primary endpoint timeframe NRS/VAS pain score and variability.
- Weekly average of daily pain and variability.
- Baseline and primary endpoint timeframe KOOS pain subscale score and variability.
- Baseline and primary endpoint timeframe PGA score and variability.
- Baseline and primary endpoint timeframe ICOAP score and variability.
- Baseline and primary endpoint timeframe ICOAP score and variability.

Prioritisation of outcomes

Efficacy measures are prioritised in descending order in section 5.6.6.

Risk of bias in individual studies

Not available.

Data synthesis

All eligible studies will be included for analysis if the available data permits it.

Data will be summarised using descriptive statistics and presented in listings, tables, and figures.

As objectives are related to variability, as opposed to estimates, data will be tabulated and illustrated. Scores will be standardised on a 0-100 scale. The association between contextual factors and the primary outcome will be analysed using linear regression.

Meta-bias(es)

Not available.

Confidence in cumulative evidence

Not available.

Ethical considerations

This study does not require approval by the ethical committee.

Dissemination

The results will, regardless of the findings, be disseminated as an article in a peer-reviewed scientific journal and will be communicated via suitable sources.

Perspective

This study will quantify the variability of pain assessment(s) in knee OA RCTs, which will help facilitate the assessment of the relative efficacies and rankings of active treatment.

Contributions

First version of this protocol was written by JB. All authors assisted in the final protocol and agreed to the final version before submission.

Support

Authors are employees at NBCD A/S. The review did not receive any specific funding or other sources of financial or other support. Only the authors influence study design, data collection, data synthesis, data interpretation, writing the report and decision to submit the manuscript.

References

1. Tuttle AH, Tohyama S, Ramsay T, Kimmelman J, Schweinhardt P, Bennett GJ, et al. Increasing placebo responses over time in U.S. clinical trials of neuropathic pain. Pain. 2015 Dec;156(12):2616–26.

2. Beaudart C, Lengelé L, Leclercq V, Geerinck A, Sanchez-Rodriguez D, Bruyère O, et al. Symptomatic Efficacy of Pharmacological Treatments for Knee Osteoarthritis: A Systematic Review and a Network Meta-Analysis with a 6-Month Time Horizon. Drugs. 2020 Dec 19;80(18):1947–59.

3. Pitz M, Cheang M, Bernstein C. Defining the predictors of the placebo response in irritable bowel syndrome. Clinical Gastroenterology and Hepatology. 2005 Mar;3(3):237–47.

4. Murphy L, Schwartz TA, Helmick CG, Renner JB, Tudor G, Koch G, et al. Lifetime risk of symptomatic knee osteoarthritis. Arthritis Care Res (Hoboken). 2008 Sep 15;59(9):1207–13.

5. Dominick KL, Ahern FM, Gold CH, Heller DA. Health-related quality of life among older adults with arthritis. Health Qual Life Outcomes. 2004;2(1):5.

6. Bellamy N, Buchanan WW, Goldsmith CH, Campbell J, Stitt LW. Validation study of WOMAC: a health status instrument for measuring clinically important patient relevant outcomes to antirheumatic drug therapy in patients with osteoarthritis of the hip or knee. J Rheumatol. 1988 Dec;15(12):1833–40.

7. Woolf AD, Pfleger B. Burden of major musculoskeletal conditions. Bull World Health Organ. 2003;81(9):646–56.

8. Yu SP, van Middelkoop M, Deveza LA, Ferreira ML, Bierma‐Zeinstra S, Zhang W, et al. Predictors of Placebo Response to Local ( <scp>Intra‐Articular</scp> ) Therapy In Osteoarthritis: An Individual Participant Data <scp>Meta‐Analysis</scp>. Arthritis Care Res (Hoboken). 2023 Oct 25;

9. Zhang W, Robertson J, Jones AC, Dieppe PA, Doherty M. The placebo effect and its determinants in osteoarthritis: meta-analysis of randomised controlled trials. Ann Rheum Dis. 2008 Dec 1;67(12):1716–23.

10. Moher D, Shamseer L, Clarke M, Ghersi D, Liberati A, Petticrew M, et al. Preferred reporting items for systematic review and meta-analysis protocols (PRISMA-P) 2015 statement. Syst Rev. 2015 Dec 1;4(1):1.

11. Ouzzani M, Hammady H, Fedorowicz Z, Elmagarmid A. Rayyan—a web and mobile app for systematic reviews. Syst Rev. 2016 Dec 5;5(1):210.

**Detailed account of the protocol registration process:**

On 04 October 2024, a final meeting was held with the affiliated information specialist from the university library, during which the definitive search strategy was agreed upon. The strategy was designed to cover the period from 2010 to present (i.e., 04 October 2024). Following consensus at this meeting, the search strategy was formally locked. The study protocol was finalised shortly thereafter and submitted for registration.

The protocol was first submitted to PROSPERO on 16 October 2024 (reference number #602233). On 27 October 2024, an automated message was received from the PROSPERO Administrator (Centre for Reviews and Dissemination) indicating that, due to substantial backlog and prioritisation of UK/NIHR-funded and COVID-19–related submissions, non-prioritised records awaiting registration for more than 10 days would be automatically processed.

After waiting one month, the protocol was resubmitted to PROSPERO on 26 November 2024 under a new reference number (#619082), which was updated in the protocol document. Ten days later, on 07 December 2024, a similar automated rejection message was received.

Because the rejection was received on a Saturday (07 December 2024), the study was subsequently registered with the Open Science Framework (OSF; <https://doi.org/10.17605/OSF.IO/YZR5J>) on the next working day, Monday, 09 December 2024. We regret that the protocol document was not updated prior to OSF registration to reflect this change in registry.

The literature search was conducted on 05 December 2024. As indicated in the PROSPERO acknowledgement email, authors were permitted to continue working on the review while awaiting registration. Accordingly, the search was performed after the protocol had been finalised, locked, and submitted to PROSPERO (twice), but prior to formal registration. Two days later, it became evident that the second PROSPERO submission would not be accepted, which resulted in OSF registration postdating the search.

Importantly, the protocol had been finalised and formally submitted before the literature search was conducted, and all procedures followed the instructions provided by PROSPERO. Although the approach was methodologically valid, we acknowledge in retrospect that updating the search end date would have improved transparency.

In summary:

- 04 October 2024: Search strategy lock.
- 16 October 2024: Protocol submission to PROSPERO (#602233).
- 26 November 2024: Protocol resubmission to PROSPERO (#619082).
- 05 December 2024: Literature search.
- 09 December 2024: Protocol submission to OSF (https://doi.org/10.17605/OSF.IO/YZR5J).

Appendix 2:

***Search strategy***

**Pubmed**

(((((("Osteoarthritis, Knee"[Mesh]) OR (knee osteoarthritis[Text Word])) OR (osteoarthritis of the knee[Text Word])) OR ("Musculoskeletal Pain"[Mesh])) AND ((("Placebos"[Mesh]) OR ("Placebo Effect"[Mesh])) OR (placebo*[Text Word]))) AND ((("Western Ontario and McMaster Universities Arthritis Index"[Text Word]) OR (WOMAC[Text Word])) OR ("Pain Measurement"[Mesh]))) AND ("Randomized Controlled Trial" [Publication Type]) Filters: from 2010 – 2024

**Embase**

('knee osteoarthritis'/exp OR 'knee osteoarthritis':ti,ab,kw OR 'osteoarthritis of the knee':ti,ab,kw OR 'musculoskeletal pain'/exp) AND ('placebo'/exp OR 'placebo effect'/exp OR placebo*:ti,ab,kw) AND ('pain measurement'/exp OR 'western ontario and mcmaster universities osteoarthritis index':ti,ab,kw OR womac:ti,ab,kw) AND 'randomized controlled trial'/exp AND [2010-2024]/py

***Search protocol***

**PubMed 04OKT2024:**

| Search # | Search term | Results |
| --- | --- | --- |
| 1 | "Osteoarthritis, Knee"[Mesh] | [29,666](https://pubmed.ncbi.nlm.nih.gov/?sort=date&term=%22Osteoarthritis%2C+Knee%22%5BMesh%5D&size=50) |
| 2 | knee osteoarthritis[Text Word] | [18,522](https://pubmed.ncbi.nlm.nih.gov/?term=knee+osteoarthritis%5BText+Word%5D&sort=date&size=50) |
| 3 | osteoarthritis of the knee[Text Word] | [3,507](https://pubmed.ncbi.nlm.nih.gov/?term=osteoarthritis+of+the+knee%5BText+Word%5D&sort=date&size=50) |
| 4 | "Musculoskeletal Pain"[Mesh] | [8,075](https://pubmed.ncbi.nlm.nih.gov/?sort=date&term=%22Musculoskeletal+Pain%22%5BMesh%5D&size=50) |
| 5 | 1 – 4 (OR)  ((("Osteoarthritis, Knee"[Mesh]) OR (knee osteoarthritis[Text Word])) OR (osteoarthritis of the knee[Text Word])) OR ("Musculoskeletal Pain"[Mesh]) | [44,571](https://pubmed.ncbi.nlm.nih.gov/?term=%28%28%28%22Osteoarthritis%2C+Knee%22%5BMesh%5D%29+OR+%28knee+osteoarthritis%5BText+Word%5D%29%29+OR+%28osteoarthritis+of+the+knee%5BText+Word%5D%29%29+OR+%28%22Musculoskeletal+Pain%22%5BMesh%5D%29&sort=date&size=50) |
| 6 | “Placebos"[Mesh] | [39,926](https://pubmed.ncbi.nlm.nih.gov/?sort=date&term=%22Placebos%22%5BMesh%5D&size=50) |
| 7 | “Placebo Effect"[Mesh] | [5,595](https://pubmed.ncbi.nlm.nih.gov/?sort=date&term=%22Placebo+Effect%22%5BMesh%5D&size=50) |
| 8 | placebo*[Text Word] | [274,987](https://pubmed.ncbi.nlm.nih.gov/?term=placebo%2A%5BText+Word%5D&sort=date&size=50) |
| 9 | 6-8 (OR)  (("Placebos"[Mesh]) OR ("Placebo Effect"[Mesh])) OR (placebo*[Text Word]) | [279,079](https://pubmed.ncbi.nlm.nih.gov/?term=%28%28%22Placebos%22%5BMesh%5D%29+OR+%28%22Placebo+Effect%22%5BMesh%5D%29%29+OR+%28placebo%2A%5BText+Word%5D%29&sort=date&size=50) |
| 10 | 5 AND 9  (((("Osteoarthritis, Knee"[Mesh]) OR (knee osteoarthritis[Text Word])) OR (osteoarthritis of the knee[Text Word])) OR ("Musculoskeletal Pain"[Mesh])) AND ((("Placebos"[Mesh]) OR ("Placebo Effect"[Mesh])) OR (placebo*[Text Word])) | [2,149](https://pubmed.ncbi.nlm.nih.gov/?term=%28%28%28%28%22Osteoarthritis%2C+Knee%22%5BMesh%5D%29+OR+%28knee+osteoarthritis%5BText+Word%5D%29%29+OR+%28osteoarthritis+of+the+knee%5BText+Word%5D%29%29+OR+%28%22Musculoskeletal+Pain%22%5BMesh%5D%29%29+AND+%28%28%28%22Placebos%22%5BMesh%5D%29+OR+%28%22Placebo+Effect%22%5BMesh%5D%29%29+OR+%28placebo%2A%5BText+Word%5D%29%29&sort=date&size=50) |
| 11 | "Pain Measurement"[Mesh] | [97,466](https://pubmed.ncbi.nlm.nih.gov/?sort=date&term=%22Pain+Measurement%22%5BMesh%5D&size=50) |
| 12 | "Western Ontario and McMaster Universities Arthritis Index"[Text Word] | [713](https://pubmed.ncbi.nlm.nih.gov/?term=%22Western+Ontario+and+McMaster+Universities+Arthritis+Index%22%5BText+Word%5D&sort=date&size=50) |
| 13 | WOMAC[Text Word] | [6,678](https://pubmed.ncbi.nlm.nih.gov/?term=WOMAC%5BText+Word%5D&sort=date&size=50) |
| 14 | 11-13 (OR)  (("Western Ontario and McMaster Universities Arthritis Index"[Text Word]) OR (WOMAC[Text Word])) OR ("Pain Measurement"[Mesh]) | [102,866](https://pubmed.ncbi.nlm.nih.gov/?term=%28%28%22Western+Ontario+and+McMaster+Universities+Arthritis+Index%22%5BText+Word%5D%29+OR+%28WOMAC%5BText+Word%5D%29%29+OR+%28%22Pain+Measurement%22%5BMesh%5D%29&sort=date&size=50) |
| 15 | 10 (AND) 14  ((((("Osteoarthritis, Knee"[Mesh]) OR (knee osteoarthritis[Text Word])) OR (osteoarthritis of the knee[Text Word])) OR ("Musculoskeletal Pain"[Mesh])) AND ((("Placebos"[Mesh]) OR ("Placebo Effect"[Mesh])) OR (placebo*[Text Word]))) AND ((("Western Ontario and McMaster Universities Arthritis Index"[Text Word]) OR (WOMAC[Text Word])) OR ("Pain Measurement"[Mesh])) | [896](https://pubmed.ncbi.nlm.nih.gov/?term=%28%28%28%28%28%22Osteoarthritis%2C+Knee%22%5BMesh%5D%29+OR+%28knee+osteoarthritis%5BText+Word%5D%29%29+OR+%28osteoarthritis+of+the+knee%5BText+Word%5D%29%29+OR+%28%22Musculoskeletal+Pain%22%5BMesh%5D%29%29+AND+%28%28%28%22Placebos%22%5BMesh%5D%29+OR+%28%22Placebo+Effect%22%5BMesh%5D%29%29+OR+%28placebo%2A%5BText+Word%5D%29%29%29+AND+%28%28%28%22Western+Ontario+and+McMaster+Universities+Arthritis+Index%22%5BText+Word%5D%29+OR+%28WOMAC%5BText+Word%5D%29%29+OR+%28%22Pain+Measurement%22%5BMesh%5D%29%29&sort=date&size=50) |
| 16 | 15 (AND) "Randomized Controlled Trial" [Publication Type]  (((((("Osteoarthritis, Knee"[Mesh]) OR (knee osteoarthritis[Text Word])) OR (osteoarthritis of the knee[Text Word])) OR ("Musculoskeletal Pain"[Mesh])) AND ((("Placebos"[Mesh]) OR ("Placebo Effect"[Mesh])) OR (placebo*[Text Word]))) AND ((("Western Ontario and McMaster Universities Arthritis Index"[Text Word]) OR (WOMAC[Text Word])) OR ("Pain Measurement"[Mesh]))) AND ("Randomized Controlled Trial" [Publication Type]) | [595](https://pubmed.ncbi.nlm.nih.gov/?term=%28%28%28%28%28%28%22Osteoarthritis%2C+Knee%22%5BMesh%5D%29+OR+%28knee+osteoarthritis%5BText+Word%5D%29%29+OR+%28osteoarthritis+of+the+knee%5BText+Word%5D%29%29+OR+%28%22Musculoskeletal+Pain%22%5BMesh%5D%29%29+AND+%28%28%28%22Placebos%22%5BMesh%5D%29+OR+%28%22Placebo+Effect%22%5BMesh%5D%29%29+OR+%28placebo%2A%5BText+Word%5D%29%29%29+AND+%28%28%28%22Western+Ontario+and+McMaster+Universities+Arthritis+Index%22%5BText+Word%5D%29+OR+%28WOMAC%5BText+Word%5D%29%29+OR+%28%22Pain+Measurement%22%5BMesh%5D%29%29%29+AND+%28%22Randomized+Controlled+Trial%22+%5BPublication+Type%5D%29&sort=date&size=50) |
| 17 | 16 (AND) Filters: from 2010 – 2024  (((((("Osteoarthritis, Knee"[Mesh]) OR (knee osteoarthritis[Text Word])) OR (osteoarthritis of the knee[Text Word])) OR ("Musculoskeletal Pain"[Mesh])) AND ((("Placebos"[Mesh]) OR ("Placebo Effect"[Mesh])) OR (placebo*[Text Word]))) AND ((("Western Ontario and McMaster Universities Arthritis Index"[Text Word]) OR (WOMAC[Text Word])) OR ("Pain Measurement"[Mesh]))) AND ("Randomized Controlled Trial" [Publication Type]) Filters: from 2010 - 2024 | [418](https://pubmed.ncbi.nlm.nih.gov/?term=%28%28%28%28%28%22Osteoarthritis%2C+Knee%22%5BMesh%5D%29+OR+%28knee+osteoarthritis%5BText+Word%5D%29%29+OR+%28osteoarthritis+of+the+knee%5BText+Word%5D%29%29+OR+%28%22Musculoskeletal+Pain%22%5BMesh%5D%29%29+AND+%28%28%28%22Placebos%22%5BMesh%5D%29+OR+%28%22Placebo+Effect%22%5BMesh%5D%29%29+OR+%28placebo%2A%5BText+Word%5D%29%29%29+AND+%28%28%28%22Western+Ontario+and+McMaster+Universities+Arthritis+Index%22%5BText+Word%5D%29+OR+%28WOMAC%5BText+Word%5D%29%29+OR+%28%22Pain+Measurement%22%5BMesh%5D%29%29&filter=pubt.randomizedcontrolledtrial&filter=years.2010-2024&sort=date&size=50) |

**Embase 04OKT2024:**

| Search # | Search term | Results |
| --- | --- | --- |
| 1 | 'knee osteoarthritis'/exp | 49,168 |
| 2 | 'knee osteoarthritis':ti,ab,kw | 28,064 |
| 3 | 'osteoarthritis of the knee':ti,ab,kw | 4,753 |
| 4 | 'musculoskeletal pain'/exp | 205,349 |
| 5 | 1 – 4 (OR)  'knee osteoarthritis'/exp OR 'knee osteoarthritis':ti,ab,kw OR 'osteoarthritis of the knee':ti,ab,kw OR 'musculoskeletal pain'/exp | 256,366 |
| 6 | 'placebo'/exp | 424,971 |
| 7 | 'placebo effect'/exp | 8,054 |
| 8 | placebo*:ti,ab,kw | 386,275 |
| 9 | 6-8 (OR)  'placebo'/exp OR 'placebo effect'/exp OR placebo*:ti,ab,kw | 551,893 |
| 10 | 5 AND 9  ('knee osteoarthritis'/exp OR 'knee osteoarthritis':ti,ab,kw OR 'osteoarthritis of the knee':ti,ab,kw OR 'musculoskeletal pain'/exp) AND ('placebo'/exp OR 'placebo effect'/exp OR placebo*:ti,ab,kw) | 14,719 |
| 11 | 'pain measurement'/exp | 36,602 |
| 12 | 'western ontario and mcmaster universities osteoarthritis index':ti,ab,kw | 3,870 |
| 13 | womac:ti,ab,kw | 11,161 |
| 14 | 11-13 (OR)  'pain measurement'/exp OR 'western ontario and mcmaster universities osteoarthritis index':ti,ab,kw OR womac:ti,ab,kw | 48,141 |
| 15 | 10 (AND) 14  ('knee osteoarthritis'/exp OR 'knee osteoarthritis':ti,ab,kw OR 'osteoarthritis of the knee':ti,ab,kw OR 'musculoskeletal pain'/exp) AND ('placebo'/exp OR 'placebo effect'/exp OR placebo*:ti,ab,kw) AND ('pain measurement'/exp OR 'western ontario and mcmaster universities osteoarthritis index':ti,ab,kw OR womac:ti,ab,kw) | 1,618 |
| 16 | 15 (AND) 'randomized controlled trial'/exp  ('knee osteoarthritis'/exp OR 'knee osteoarthritis':ti,ab,kw OR 'osteoarthritis of the knee':ti,ab,kw OR 'musculoskeletal pain'/exp) AND ('placebo'/exp OR 'placebo effect'/exp OR placebo*:ti,ab,kw) AND ('pain measurement'/exp OR 'western ontario and mcmaster universities osteoarthritis index':ti,ab,kw OR womac:ti,ab,kw) AND 'randomized controlled trial'/exp | 1,047 |
| 17 | 16 (AND) [2010-2024]/py  ('knee osteoarthritis'/exp OR 'knee osteoarthritis':ti,ab,kw OR 'osteoarthritis of the knee':ti,ab,kw OR 'musculoskeletal pain'/exp) AND ('placebo'/exp OR 'placebo effect'/exp OR placebo*:ti,ab,kw) AND ('pain measurement'/exp OR 'western ontario and mcmaster universities osteoarthritis index':ti,ab,kw OR womac:ti,ab,kw) AND 'randomized controlled trial'/exp AND [2010-2024]/py | [900](https://pubmed.ncbi.nlm.nih.gov/?term=%28%28%28%28%28%22Osteoarthritis%2C+Knee%22%5BMesh%5D%29+OR+%28knee+osteoarthritis%5BText+Word%5D%29%29+OR+%28osteoarthritis+of+the+knee%5BText+Word%5D%29%29+OR+%28%22Musculoskeletal+Pain%22%5BMesh%5D%29%29+AND+%28%28%28%22Placebos%22%5BMesh%5D%29+OR+%28%22Placebo+Effect%22%5BMesh%5D%29%29+OR+%28placebo%2A%5BText+Word%5D%29%29%29+AND+%28%28%28%22Western+Ontario+and+McMaster+Universities+Arthritis+Index%22%5BText+Word%5D%29+OR+%28WOMAC%5BText+Word%5D%29%29+OR+%28%22Pain+Measurement%22%5BMesh%5D%29%29&filter=pubt.randomizedcontrolledtrial&filter=years.2010-2024&sort=date&size=50) |

Appendix 3:

Exclusion by reason after full-text screening:

| **Authors** | **Year** | **Title** | **Exclusion reason** |
| --- | --- | --- | --- |
| Ross | 2024 | The effect of intra-articular autologous protein solution on knee osteoarthritis symptoms. | wrong outcome |
| Yoshioka | 2024 | The Effectiveness of Leukocyte-Poor Platelet-Rich Plasma Injections for Symptomatic Mild to Moderate Osteoarthritis of the Knee With Joint Effusion or Bone Marrow Lesions in a Japanese Population: A Randomized, Double-Blind, Placebo-Controlled Clinical Trial. | wrong outcome |
| Kingsbury | 2024 | Pain Reduction With Oral Methotrexate in Knee Osteoarthritis : A Randomized, Placebo-Controlled Clinical Trial. | wrong outcome |
| Aiad | 2024 | Metformin as adjuvant therapy in obese knee osteoarthritis patients. | wrong drug |
| Hill | 2023 | A Multicenter, Randomized, Double-Blinded, Placebo-Controlled Clinical Trial to Evaluate the Efficacy and Safety of a Krill Oil, Astaxanthin, and Oral Hyaluronic Acid Complex on Joint Health in People with Mild Osteoarthritis. | wrong drug |
| Möller | 2023 | Randomized, double-blind, placebo-controlled study to evaluate the effect of treatment with an SPMs-enriched oil on chronic pain and inflammation, functionality, and quality of life in patients with symptomatic knee osteoarthritis: GAUDI study. | wrong outcome |
| Gupta | 2023 | Efficacy and Safety of Stempeucel in Osteoarthritis of the Knee: A Phase 3 Randomized, Double-Blind, Multicenter, Placebo-Controlled Study. | wrong outcome |
| Tschopp | 2023 | A Randomized Trial of Intra-articular Injection Therapy for Knee Osteoarthritis. | wrong outcome |
| Yazici | 2021 | A Phase 2b randomized trial of lorecivivint, a novel intra-articular CLK2/DYRK1A inhibitor and Wnt pathway modulator for knee osteoarthritis. | wrong outcome |
| Tucker | 2021 | Randomized, Placebo-Controlled Analysis of the Knee Synovial Environment Following Platelet-Rich Plasma Treatment for Knee Osteoarthritis. | wrong outcome |
| Fraenkel | 2020 | Nonsteroidal Anti-inflammatory Drugs vs Cognitive Behavioral Therapy for Arthritis Pain: A Randomized Withdrawal Trial. | wrong outcome |
| Garza | 2020 | Clinical Efficacy of Intra-articular Mesenchymal Stromal Cells for the Treatment of Knee Osteoarthritis: A Double-Blinded Prospective Randomized Controlled Clinical Trial. | wrong outcome |
| Steels | 2019 | A double-blind randomized placebo controlled study assessing safety, tolerability and efficacy of palmitoylethanolamide for symptoms of knee osteoarthritis. | wrong outcome |
| Stevens | 2019 | Randomized, Double-Blind, Placebo-Controlled Trial of Intraarticular Trans-Capsaicin for Pain Associated With Osteoarthritis of the Knee. | wrong outcome |
| Lee | 2019 | Intra-Articular Injection of Autologous Adipose Tissue-Derived Mesenchymal Stem Cells for the Treatment of Knee Osteoarthritis: A Phase IIb, Randomized, Placebo-Controlled Clinical Trial. | wrong outcome |
| Shrestha | 2018 | Clinical Outcome following Intra-articular Triamcinolone Injection in Osteoarthritic Knee at the Community: A Randomized Double Blind Placebo Controlled Trial. | wrong outcome |
| Lin | 2019 | Intra-articular Injection of Platelet-Rich Plasma Is Superior to Hyaluronic Acid or Saline Solution in the Treatment of Mild to Moderate Knee Osteoarthritis: A Randomized, Double-Blind, Triple-Parallel, Placebo-Controlled Clinical Trial. | wrong outcome |
| Enomoto | 2018 | Efficacy of duloxetine by prior NSAID use in the treatment of chronic osteoarthritis knee pain: A post hoc subgroup analysis of a randomized, placebo-controlled, phase 3 study in Japan. | wrong outcome |
| Tétreault | 2018 | Inferring distinct mechanisms in the absence of subjective differences: Placebo and centrally acting analgesic underlie unique brain adaptations. | wrong outcome |
| Mayorga | 2017 | A randomized study to evaluate the analgesic efficacy of a single dose of the TRPV1 antagonist mavatrep in patients with osteoarthritis. | wrong outcome |
| Lubis | 2017 | Comparison of Glucosamine-Chondroitin Sulfate with and without Methylsulfonylmethane in Grade I-II Knee Osteoarthritis: A Double Blind Randomized Controlled Trial. | wrong outcome |
| Moss | 2017 | Fourteen days of etoricoxib 60 mg improves pain, hyperalgesia and physical function in individuals with knee osteoarthritis: a randomized controlled trial. | wrong outcome |
| Strand | 2017 | Low-dose SoluMatrix diclofenac in patients with osteoarthritis pain: impact on quality of life in a controlled trial. | wrong publication type |
| Arendt-Nielsen | 2017 | Intra-articular onabotulinumtoxinA in osteoarthritis knee pain: effect on human mechanistic pain biomarkers and clinical pain. | wrong outcome |
| Arendt-Nielsen | 2016 | Evidence for a central mode of action for etoricoxib (COX-2 inhibitor) in patients with painful knee osteoarthritis. | wrong outcome |
| Smith | 2016 | Intra-articular Autologous Conditioned Plasma Injections Provide Safe and Efficacious Treatment for Knee Osteoarthritis: An FDA-Sanctioned, Randomized, Double-blind, Placebo-controlled Clinical Trial. | wrong outcome |
| Gow | 2015 | Safety, tolerability, pharmacokinetics, and efficacy of AMG 403, a human anti-nerve growth factor monoclonal antibody, in two phase I studies with healthy volunteers and knee osteoarthritis subjects. | wrong outcome |
| Holt | 2015 | Onset and durability of pain relief in knee osteoarthritis: Pooled results from two placebo trials of naproxen/esomeprazole combination and celecoxib. | wrong outcome |
| Symonds | 2015 | Validation of the Chinese Western Ontario and McMaster Universities Osteoarthritis Index in Patients From Mainland China With Osteoarthritis of the Knee. | wrong outcome |
| Varenna | 2015 | Intravenous neridronate in the treatment of acute painful knee osteoarthritis: a randomized controlled study. | wrong outcome |
| van | 2015 | No difference between intra-articular injection of hyaluronic acid and placebo for mild to moderate knee osteoarthritis: a randomized, controlled, double-blind trial. | wrong outcome |
| Rossini | 2015 | Effects of intra-articular clodronate in the treatment of knee osteoarthritis: results of a double-blind, randomized placebo-controlled trial. | wrong outcome |
| Schnitzer | 2015 | Efficacy and safety of tanezumab monotherapy or combined with non-steroidal anti-inflammatory drugs in the treatment of knee or hip osteoarthritis pain. | wrong drug |
| Trudeau | 2015 | Assessment of pain and activity using an electronic pain diary and actigraphy device in a randomized, placebo-controlled crossover trial of celecoxib in osteoarthritis of the knee. | wrong outcome |
| Abou-Raya | 2014 | Effect of low-dose oral prednisolone on symptoms and systemic inflammation in older adults with moderate to severe knee osteoarthritis: a randomized placebo-controlled trial. | retracted |
| Balanescu | 2014 | Efficacy and safety of tanezumab added on to diclofenac sustained release in patients with knee or hip osteoarthritis: a double-blind, placebo-controlled, parallel-group, multicentre phase III randomised clinical trial. | wrong drug |
| Risser | 2013 | Responsiveness of the Intermittent and Constant Osteoarthritis Pain (ICOAP) scale in a trial of duloxetine for treatment of osteoarthritis knee pain. | wrong outcome |
| Patel | 2013 | Treatment with platelet-rich plasma is more effective than placebo for knee osteoarthritis: a prospective, double-blind, randomized trial. | wrong outcome |
| Abou-Raya | 2012 | Duloxetine for the management of pain in older adults with knee osteoarthritis: randomised placebo-controlled trial. | wrong outcome |
| Huggins | 2012 | An efficient randomised, placebo-controlled clinical trial with the irreversible fatty acid amide hydrolase-1 inhibitor PF-04457845, which modulates endocannabinoids but fails to induce effective analgesia in patients with pain due to osteoarthritis of the knee. | wrong outcome |
| Singh | 2012 | Diacerein as adjuvant to diclofenac sodium in osteoarthritis knee. | wrong drug |
| Boyer | 2012 | Sensitivity of gait parameters to the effects of anti-inflammatory and opioid treatments in knee osteoarthritis patients. | wrong outcome |
| Navarro-Sarabia | 2011 | A 40-month multicentre, randomised placebo-controlled study to assess the efficacy and carry-over effect of repeated intra-articular injections of hyaluronic acid in knee osteoarthritis: the AMELIA project. | wrong outcome |
| Cohen | 2011 | A randomized, double-blind study of AMG 108 (a fully human monoclonal antibody to IL-1R1) in patients with osteoarthritis of the knee. | wrong outcome |
| Hochberg | 2011 | Fixed-dose combination of enteric-coated naproxen and immediate-release esomeprazole has comparable efficacy to celecoxib for knee osteoarthritis: two randomized trials. | wrong outcome |
| Hunter | 2010 | Phase 1 safety and tolerability study of BMP-7 in symptomatic knee osteoarthritis. | wrong outcome |
| Chappell | 2011 | A double-blind, randomized, placebo-controlled study of the efficacy and safety of duloxetine for the treatment of chronic pain due to osteoarthritis of the knee. | wrong outcome |
| Sawitzke | 2010 | Clinical efficacy and safety of glucosamine, chondroitin sulphate, their combination, celecoxib or placebo taken to treat osteoarthritis of the knee: 2-year results from GAIT. | wrong outcome |
| Frampton | 2010 | Hylan G-F 20 single-injection formulation. | wrong outcome |
| Richter, | 2024 | Microfragmented Adipose Tissue Injection Reduced Pain Compared With a Saline Control Among Patients With Symptomatic Osteoarthritis of the Knee During 1-Year Follow-Up: A Randomized Controlled Trial | wrong outcome |
| Youn, | 2023 | Efficacy and safety of diacerein and celecoxib combination therapy for knee osteoarthritis: A double-blind, randomized, placebo-controlled prospective study | wrong drug |
| Saraf, | 2023 | Serial intraarticular injections of growth factor concentrate in knee osteoarthritis: A placebo controlled randomized study | wrong outcome |
| Choudharay, | 2023 | Platelet Rich Plasma Intraarticular Injections for Knee Osteoarthritis: A Prospective Study | wrong outcome |
| Petersen, | 2022 | The effect of duloxetine on mechanistic pain profiles, cognitive factors and clinical pain in patients with painful knee osteoarthritis—A randomized, double-blind, placebo-controlled, crossover study | wrong outcome |
| Mease, | 2021 | Efficacy of subcutaneous tanezumab for the treatment of osteoarthritis of the knee or hip: A post Hoc subgroup analysis of patients from a randomized, NSAID-controlled study with a history of depression, anxiety, or insomnia | wrong publication type |
| Jasim, | 2021 | The effect of atorvastatin in knee osteoarthritis | wrong outcome |
| Bhatia, | 2020 | Evaluation of efficacy and safety of a novel lipogel containing diclofenac: A randomized, placebo controlled, double-blind clinical trial in patients with signs and symptoms of osteoarthritis | wrong outcome |
| Dehghan, | 2020 | Comparison of the effect of topical Hedera helix L. extract gel to diclofenac gel in the treatment of knee osteoarthritis | wrong outcome |
| Enteshari-Moghaddam, | 2019 | Efficacy of methotrexate on pain severity reduction and improvement of quality of life in patients with moderate to severe knee osteoarthritis | wrong outcome |
| Pereira, | 2019 | Gait analysis following single-shot hyaluronic acid supplementation: A pilot randomized double-blinded controlled trial | wrong outcome |
| Yue, | 2019 | Clinical meaningfulness of duloxetine’s effect in chinese Patients with chronic pain due to osteoarthritis: Post hoc analyses of a phase 3 randomized trial | wrong outcome |
| Yu, | 2018 | Clinical therapy of hyaluronic acid combined with platelet-rich plasma for the treatment of knee osteoarthritis | wrong outcome |
| Kim, | 2018 | A Multicenter, Double-Blind, Phase III Clinical Trial to Evaluate the Efficacy and Safety of a Cell and Gene Therapy in Knee Osteoarthritis Patients | wrong outcome |
| Amirpour, | 2016 | The effect of colchicine in improving the symptoms of patients with knee osteoarthritis | wrong outcome |
| Zhao, | 2016 | Therapeutic effects of low-frequency phonophoresis with a Chinese herbal medicine versus sodium diclofenac for treatment of knee osteoarthritis: a double-blind, randomized, placebo-controlled clinical trial | wrong drug |
| Bolten, | 2015 | The safety and efficacy of an enzyme combination in managing knee osteoarthritis pain in adults: A randomized, double-blind, placebo-controlled trial | wrong outcome |
| Wonggokusuma, | 2014 | Effects of glucosamine-chondroitin sulfate, glucosamine-chondroitin sulfate-methylsulfonylmethane, or placebo in patients with first and second grade of knee osteoarthritis: A double blind randomized controlled study | wrong outcome |
| Sadeghi, | 2014 | Low dose glucosamine and chondroitin sulfate use in knee osteoarthritis | wrong outcome |
| Jensen, | 2014 | The reciprocal effects of pain intensity and activity limitations: Implications for outcomes assessment in clinical trials | wrong outcome |
| Jokar, | 2013 | The effect of hydroxychloroquine on symptoms of knee osteoarthritis: A double-blind randomized controlled clinical trial | wrong outcome |
| Sanga, | 2013 | Efficacy, safety, and tolerability of fulranumab, an anti-nerve growth factor antibody, in the treatment of patients with moderate to severe osteoarthritis pain | wrong outcome |
| Erhan, | 2012 | The efficacy of topical glucosamine sulfate-chondroitin sulfate in knee osteoarthritis treated with physical therapy: A randomized, double-blind, placebo-controlled study | wrong outcome |
| Bingham | 2011 | Predictors of Response to Cyclo-Oxygenase-2 Inhibitors in Osteoarthritis: Pooled Results from Two Identical Trials Comparing Etoricoxib, Celecoxib, and Placebo | wrong outcome |
| Jokar, | 2010 | The effect of alendronate on symptoms of knee osteoarthritis: A randomized controlled trial | wrong outcome |
| Berenbaum | 2021 | Subcutaneous tanezumab for osteoarthritis: Is the early improvement in pain and function meaningful and sustained? | wrong publication type |
| Sadeghi | 2014 | Strontium ranolate effect on knee osteoarthritis | wrong outcome |
| Schnitzer | 2020 | Onset and maintenance of efficacy of subcutaneous tanezumab in patients with moderate to severe osteoarthritis of the knee or hip: A 16-week dose-titration study | previously reported |
| Wu | 2018 | Effects of Platelet-Rich Plasma on Pain and Muscle Strength in Patients With Knee Osteoarthritis | wrong outcome |

Appendix 4:

Funnel plot
